# Designing the optimal bit: balancing energetic cost, speed and reliability

**DOI:** 10.1098/rspa.2017.0117

**Published:** 2017-08-23

**Authors:** Abhishek Deshpande, Manoj Gopalkrishnan, Thomas E. Ouldridge, Nick S. Jones

**Affiliations:** 1 Department of Mathematics, Imperial College London, London SW7 2AZ, UK; 2 School of Technology and Computer Science, Tata Institute of Fundamental Research, Mumbai 400005, India; 3 Department of Electrical Engineering, Indian Institute of Technology Bombay, Powai, Mumbai 400076, India; 4 Department of Bioengineering, Imperial College London, London SW7 2AZ, UK

**Keywords:** erasing/switching a bit, particle in a double well, reliability of information, optimal bit, friction trade-off, saturation/unsaturation of time scales

## Abstract

We consider the challenge of operating a reliable bit that can be rapidly erased. We find that both erasing and reliability times are non-monotonic in the underlying friction, leading to a trade-off between erasing speed and bit reliability. Fast erasure is possible at the expense of low reliability at moderate friction, and high reliability comes at the expense of slow erasure in the underdamped and overdamped limits. Within a given class of bit parameters and control strategies, we define ‘optimal’ designs of bits that meet the desired reliability and erasing time requirements with the lowest operational work cost. We find that optimal designs always saturate the bound on the erasing time requirement, but can exceed the required reliability time if critically damped. The non-trivial geometry of the reliability and erasing time scales allows us to exclude large regions of parameter space as suboptimal. We find that optimal designs are either critically damped or close to critical damping under the erasing procedure.

## 1. Introduction

Certain information-processing operations such as erasing a bit or copying the state of one bit into another previously randomized bit have fundamental lower bounds on work input [1–5]. These lower bounds such as the famous $k_{B}T\ln2$ minimal cost for erasing arise due to equilibrium thermodynamics: there is a need to compensate for any entropy reduction in the information-carrying system with an entropy increase elsewhere. Practical devices, however, do not approach these bounds [6,7] and insights gained from thinking about the lower bound have not yet translated into more energy-efficient technology. A partial explanation is that man-made devices and biological cells need to operate on fast time scales and hence cannot involve the quasi-static manipulations necessary to reach lower bounds [8,9]. An alternative suggestion from von Neumann is that the need to store information for long periods of time (reliability) leads to high-cost architectures [10]. We explore the interplay between reliability, speed and the energetic cost of bit operation. Equilibrium thermodynamic bounds such as the Landauer limit cannot account for these inherently kinetic phenomena.

This general question of how to design fast, cheap and reliable bits has obvious technological relevance to the optimal design of low-power computational devices [11–13]. Additionally, since the discovery of the structure of DNA and the central dogma of molecular biology, it has become well accepted that information processing is at the heart of many natural phenomena. Many authors have explored information processing in biological systems, to both understand natural examples and design synthetic analogues [3,9,14–19]. The question of the interplay between reliability, speed and cost are also relevant here, although under-explored.

In this paper, we explore the challenge of building fast, cheap and reliable bits, and provide a framework for its analysis in terms of reliability and erasure time scales. We also take the first steps towards exploring the physics of the optimal design problem by considering a simple model: a particle in a one-dimensional potential, which is a quartic double-well potential in the device’s ‘resting’ state. We require that the bit be reliable, so that a particle equilibrated in either well stays in that well for a specified long time on average. Simultaneously, we require the implementation of an ‘erase’ or ‘reset’ operation using an external control, so that erasure is completed within a specified short amount of time. Our principal question is to find values for the design parameters which consist of the height of the double well, the friction coefficient, and the control parameters to guarantee these requirements without expending more energy than required. Our main contribution is an exploration of this design space, which demonstrates the previously under-appreciated role of friction. In particular, we identify a ‘Goldilocks zone’ where the friction coefficient takes moderate values. This is somewhat counter-intuitive because historically friction has been viewed as a nuisance to computing, to be sent as low as possible [20–23].

In §2, we describe the model which will provide intuition for our work. We formalize the time scale over which the bit stores information through the notion of *reliability time*. In §2b(i), we describe one simple family of control protocols for resetting a bit. We calculate the work done in erasing a bit for this form of control. We will use this particular control protocol to illustrate our subsequent ideas. In §2b(ii), we introduce the notion of *erasing time*. In §3, we consolidate from the literature the analytical forms and approximations for our two time scales of interest, and confirm them with numerical simulations. We find that both the reliability and erasing time scales are non-monotonic, roughly U-shaped functions of the friction coefficient. It follows that high reliability is obtained by setting the friction to a low or high value, whereas a low erasing time is favoured by an intermediate value of friction, implying a conflict between the two time scales for a given class of protocols. In §4, we investigate how this conflict feeds into the geometry of *optimal bits*: bits that fulfil the desired reliability and erasing time requirements with the minimum energy cost. We find and partially characterize a ‘Goldilocks zone’ in design space where optimal bits reside. In §5, we discuss the robustness of our results when more freedom is allowed in the choice of design parameters and the control protocol.

## 2. The double-well bit

**Figure 1.**
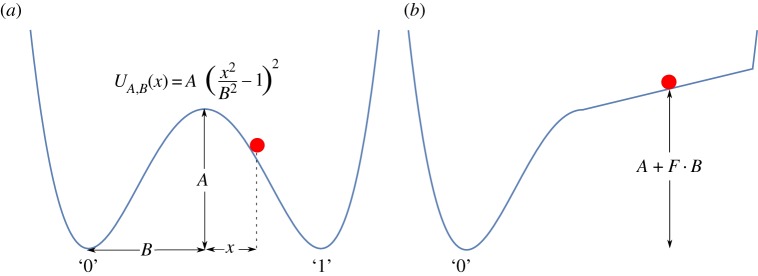
A bit as represented by a particle in a one-dimensional potential. (*a*) The bit in its resting state, with a barrier of height ‘A’ separating particle locations that correspond to bit values of 0 or 1. (*b*) A control potential as in example 2.1 is applied to erase the stored data.

We will represent a device that can store one bit of information by a particle in a symmetric bistable potential *U*_*A*,*B*_(*x*)=*A*(*x*^2^/*B*^2^−1)^2^, where *A* is the height of the well and ±*B* are the coordinates of the minima of the right and left wells. We will refer to the device as a whole as ‘a bit’. The device reports ‘0’ when the particle is in the left well, i.e. *x*<0, and reports ‘1’ otherwise (figure 1*a*).

The dynamics of the particle is described by the Langevin equation

2.1 $$\begin{array}{rl} m\;dx & =p\;dt\\ \text{and}\;dp & =-\gamma p\;dt-\partial_{x}U_{A,B}(x)\;dt+\sqrt{2m\gamma k_{B}T}\;dW, \end{array}\}$$

where *m* is the mass of the particle, *x* is the position, *p* is the momentum, *γ* is the friction coefficient of the medium, *U*_*A*,*B*_(*x*) is the potential, *k*_B_ is Boltzmann’s constant and *T* is the temperature of the heat bath. The term $\sqrt{2m\gamma k_{B}T}\;dW$ represents the effect of noise from the surroundings. The Langevin equation is a stochastic differential equation, to be mathematically interpreted as a Stratonovich integral. For our case, both the Ito and Stratonovich interpretations coincide [24], p. 109 as the noise coefficient $\sqrt{2m\gamma k_{B}T}$ does not depend upon *p*.

In [25], p. 182, the generator for the Langevin equation (2.1) is

2.2 $$L=\frac{p}{m}\partial_{x}-(\partial_{x}U_{A,B}(x))\partial_{p}+\gamma (-p\partial_{p}+k_{B}T\partial_{p}^{2}).$$

The *Hamiltonian* of the system is *H*(*x*,*p*)=*U*_*A*,*B*_(*x*)+*p*^2^/2*m*. The *Gibbs distribution*

2.3 $$\pi (x,p)=\frac{e^{-H(x,p)/k_{B}T}}{\int_{-\infty }^{\infty }\int_{-\infty }^{\infty }e^{-H(x^{\prime },p^{\prime })/k_{B}T}\;dx^{\prime }\;dp^{\prime }}$$

is approached as the system relaxes to equilibrium. Convergence to *π*(*x*,*p*) happens exponentially fast at a rate given by the first non-zero eigenvalue of the generator L [26].

### (a) Reliability

A device to store information should be able to store it with high fidelity for a specified long period of time. We introduce the *reliability time* to represent the time scale over which our device can store data. Specifically, we define the reliability time *τ*_r_ as the expected first passage time for the particle to cross the barrier of the resting-state potential of the bit, given the Gibbs distribution *π*(*x*,*p*) (equation (2.3)) as the initial distribution. That is,

2.4 $$\tau_{r}:=E[\inf\{t\geq 0|x(t)=0\}],$$

where the expectation is over trajectories (*x*(*t*),*p*(*t*)) distributed as specified by equation (2.1) from the initial condition (*x*(0),*p*(0))∼_law_*π*(*x*,*p*). Note that *τ*_r_ is also the first passage time to cross the barrier for a bit prepared with a Gibbs distribution, but confined to either the left-hand well *π*_0_(*x*,*p*) or the right-hand well *π*_1_(*x*,*p*),

2.5 $$\pi_{0}(x,p)=\left\{ \begin{array}{ll} 2\pi (x,p) & \mathrm{if}\;x<0,\\ 0 & \mathrm{otherwise}, \end{array}\right.\;\mathrm{and}\;\pi_{1}(x,p)=\left\{ \begin{array}{ll} 2\pi (x,p) & \mathrm{if}\;x>0,\\ 0 & \mathrm{otherwise}. \end{array}\right.$$

Intuitively, once a typical particle has had enough time to reach the top of the barrier, the data stored are no longer reliable.

### (b) Setting information

A device intended to store information must provide functionality to load, or set, this information into the device. Setting information is a two-bit operation. A common use case is when a reference bit and the bit to be set are initially at some arbitrary values. We require that after the SET operation the reference bit is unchanged, whereas the bit to be set now holds a copy of the reference bit. This is the operation that Szilard [1] refers to as ‘copying’ (by contrast, Landauer [2,27] chooses to reserve the word ‘copying’ for the operation where the bit to be set is initially already known to be in the state ‘0’).

Note that in the operation of setting information, or copying in the sense of Szilard, initially the two bits are uncorrelated and unknown, whereas after the operation they are still unknown but correlated. Thus implementing this operation requires decreasing the entropy of the system. Since it is easier to study a one-bit system rather than a two-bit system, we will investigate a one-bit proxy for the task of decreasing the entropy of the system, which is the task of *erasing* a bit.

Erasing involves taking a device whose initial state is maximally unknown into a known reference state, usually ‘0’. Somewhat counter-intuitively, given the name, erasing increases the information we know about the system. What is erased is not information but randomness. It helps to keep in mind the example of erasing a blackboard where some random state with chalk marks is reset to the ‘all clear’ state.

#### (i) Erasing

The example that follows describes a simple family of control potentials to implement the erasing operation for our device, which will form the basis of our analysis. One control potential from this family is illustrated in figure 1*b*. We chose such a simple class of controls to make a full understanding feasible, setting a framework for analysing more complex protocols. We also note that arbitrary variation of a physical potential in reality is highly non-trivial; experimental studies in which complex time-dependent potentials have been applied in fact use highly dissipated mechanisms to generate ‘effective’ potentials [28,29].

**Example 2.1**

Our control potentials are described by a single parameter $F\in R_{>0}$ as follows:

2.6 $$V_{F}(x):=\left\{ \begin{array}{ll} A+F\cdot x-U_{A,B}(x) & \text{if }x\geq 0\;\mathrm{and}\;A-U_{A,B}(x)+F\cdot x\geq 0,\\ 0 & \text{otherwise.} \end{array}\right.$$

The Langevin equation in the presence of a control is

2.7 $$\begin{array}{rl} m\;dx & =p\;dt,\\ dp & =-\gamma p\;dt-\partial_{x}U_{A,B}(x)\;dt-\partial_{x}V_{F}(x)\;dt+\sqrt{2m\gamma k_{B}T}\;dW. \end{array}\}$$

Note that the control potential, as defined, is not differentiable at the boundary of the region in which it is non-zero. In practice, we assume that ∂_*x*_*V*_*F*_ changes rapidly but continuously in a small vicinity around these points.

In this work, we will consider variation of *A*, *F* and *γ* at fixed *m*, *B* and *T*, respectively. In this case, *m* specifies the natural mass scale, *B* the natural length scale and *k*_B_*T* the natural energy scale; the natural time scale is then $\sqrt{mB^{2}/k_{B}T}$. Henceforth, all numerical quantities will be reported using reduced units defined with respect to these natural scales, although *m*, *B* and *k*_B_*T* will be retained within formulae.

#### (ii) Operational view of erasing

The speed of bit operations is of practical importance: a useful bit must be reliable on much larger time scales than those required to set or switch it. The control is switched on at time 0 and switched off at an appropriately chosen time *τ*. The time *τ* is chosen beforehand, and does not depend on details of individual trajectories—a trajectory-dependent control would require measurement and feedback that itself would need accounting for [30–35]. We could declare erasing as completed and switch off the control as soon as a majority of the trajectories are expected to be in the left well. However, many of these ‘erased’ bits would have high energies compared with typical bits drawn from the equilibrium distribution in the left well, *π*_0_(*x*,*p*). Thus, they could rapidly return to the right well after a very short stay in the left well. So we insist on a more stringent condition. We require that the time *τ* should be large enough so that the majority of bits are in the target well, with an expected next passage time close to the reliability time.

One way to guarantee that the next passage time is high is by insisting on mixing, in the sense that the initial distribution *π*(*x*,*p*) comes close to a distribution of particles thermalized in the left-hand well, *π*_0_(*x*,*p*). If this happens, we can guarantee that the expected next passage time will be equal to, or close to, the expected first passage time. However, we found this criterion too stringent for the following reason. At the end of the erasing protocol, it is not necessary that the distribution is close to *π*_0_(*x*,*p*)—only that the particles tend to relax to this distribution much faster than they cross back into the right-hand well, and thus they have barrier passage times representative of particles initialized with *π*_0_(*x*,*p*). Nonetheless, we show in the electronic supplementary material, §2.1, that using such a criterion preserves the qualitative features reported below (in particular, the scaling of the erasure time with friction in the high and low friction limits).

Instead, we define an *erasure region* in well ‘0’ as all points (*x*,*p*) with total energy *H*(*x*,*p*)≤*A*−3*k*_B_*T*, where *A* is the barrier height. We look for the average first passage time to reach the erasure region for particles initiated in well ‘1’ and take this quantity to be representative of the erasing time scale. The choice of the 3*k*_B_*T* criterion is somewhat arbitrary, but has been used before by Vega *et al.* [36] to study atom-surface diffusion. As we show in the electronic supplementary material, §2.2, using 4*k*_B_*T* makes no qualitative difference to our conclusions. This metric has the merit that it provides a clear computable criterion for erasing. Below, we demonstrate that particles within the 3*k*_B_*T* erasure region do indeed have expected next passage times close to the reliability time, as required.

For a range of well parameters, we used the Langevin *A* algorithm from [37] (see the electronic supplementary material, §1, for integrator set-up and validation) to estimate *τ*(*x*,*p*), the average barrier crossing time for particles initialized at position *x* with momentum *p* in the left well, for a grid of points (*x*,*p*). The average reliability time for a given well can be approximated in terms of *τ*(*x*,*p*) as follows:

2.8 $$\tau_{r}\approx \frac{\sum_{x,p}\tau (x,p)\;e^{-H(x,p)/k_{B}T}}{\sum_{x,p}\;e^{-H(x,p)/k_{B}T}}.$$

**Figure 2.**
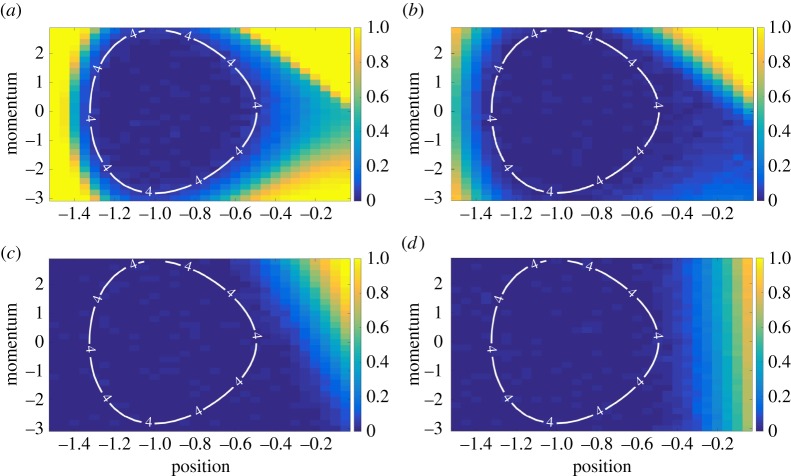
For particles initiated with *H*(*x*,*p*)≤*A*−3*k*_B_*T*, well escape times are close to *τ*_r_. Heat maps show the fractional deviation in expected escape time *δ*(*x*,*p*) from the well-thermalized average *τ*_r_, as a function of initial position *x* and momentum *p*. The labelled contours correspond to a well height *A*=7 with energy *H*(*x*,*p*)=*A*−3*k*_B_*T*=4*k*_B_*T*. These heat maps are representative of the situation for other barrier heights *A*≥5*k*_B_*T*. (*a*) *δ*(*x*,*p*) when *γ*=0.1, (*b*) *δ*(*x*,*p*) when *γ*=1, (*c*) *δ*(*x*,*p*) when *γ*=10 and (*d*) *δ*(*x*,*p*) when *γ*=100.

The deviation *δ*(*x*,*p*):=|1−*τ*(*x*,*p*)/*τ*_r_| for every point (*x*,*p*) in the grid is plotted in figure 2, for a range of friction parameters at well height *A*=7. It is clear that, for all values of friction, the points with total energy *H*(*x*,*p*)≤*A*−3*k*_B_*T* have reliability times close to *τ*_r_. The same is true of other well heights *A*. This is because such particles typically undergo thermal mixing before they can escape the well. Once mixed, their next escape over the barrier will be on a time scale of the order of *τ*_r_.

Despite the robustness of this result to the value of the friction, the heatmaps in figure 2 are friction dependent. When *γ* is low, the particle diffuses very slowly in energy space, and it is the challenge of diffusing within this energy space that prohibits escape from the well. As a result the heatmap corresponding to *γ*=0.1 (figure 2*a*) follows the shape of constant energy contours. As friction starts increasing (e.g. in figure 2*b*,*c*), diffusion in momentum space becomes more rapid, but diffusion in position space slows down. Once *γ* becomes very high (e.g. *γ*=100 in figure 2*d*), the behaviour of the heatmap is essentially determined by the initial position of the particle; those close to the barrier and with *U*_*A*,*B*_(*x*) sufficiently close to *A* can escape easily, but the momentum is irrelevant. Using the total energy *H*(*x*,*p*) as a criterion ensures that we account for all the regimes of friction.

Since we are interested in the typical time scale of transferring particles to a different well from the existent well, we will sample initial points only from the right well. We define the *erasing time* *τ*_e_ as the expected time to hit the erasure region, given that the particle started in the right-hand well:

2.9 $$\tau_{e}=E[\inf\{t\geq 0|x(t)<0\text{ and }H(x(t),p(t))\leq A-3k_{B}T\}],$$

where (*x*(*t*),*p*(*t*)) is the solution to equation (2.7) with the initial condition (*x*(0),*p*(0))∼_law_*π*_1_(*x*,*p*). Given this definition, *τ*_e_ indicates a typical time scale over which the control must be applied to successfully erase a large fraction of the bits. In practice, the control would be applied for a period *τ*>*τ*_e_ to achieve high accuracy. We will use *τ*_e_ as an indicative time scale of the control operation for the purposes of our analysis. It is useful to decompose the erasing time *τ*_e_ as the sum of two times: the *transport time* and the *mixing time*.

— *Transport time (*τ*_*t*_)*. The time taken by the particle to reach well ‘0’ given that it is initially distributed according to *π*_1_(*x*,*p*),
  2.10 $$\tau_{t}=E[\inf\{t\geq 0|x(t)\leq 0\}],$$
  where *x*(*t*) is the solution to equation (2.7) with the initial condition (*x*(0),*p*(0))∼_law_*π*_1_(*x*,*p*).
— *Mixing time (*τ*_*m*_)*. The time taken by the particle to mix sufficiently inside the well. This is the time starting from when the particle first reaches well ‘0’ to when it first hits the erasure region,
  2.11 $$\tau_{m}=\tau_{e}-\tau_{t}.$$

#### (iii) Cost of erasing

In this section, we calculate the work done in erasing a bit. From Sekimoto’s expression [38,39], for a protocol applied for a time *τ* and with a region of effect *I*={*x*≥0|*A*−*U*_*A*,*B*_(*x*)+*F*⋅*x*≥0},

2.12 $$\langle W\rangle :=\int_{0}^{\tau }\int_{x\in I}\frac{\partial V_{F}(x,t)}{\partial t}p(x,t)\;dx\;dt,$$

where *p*(*x*,*t*) *dx* *dt* is the probability that the particle is between position (*x*,*x*+*dx*) in the time interval (*t*,*t*+*dt*). There are two potential sources of work that appear in our calculation.

1. When we begin the erasure protocol by switching on the control to lift the particle.
2. At the end of the protocol when we switch off the control.

We note that, in our family of controls, there is negligible energy recovered when the control is switched off (see electronic supplementary material, §3.2), since the probability of the particle being in the region in which the control is applied is small. More generally, the question of whether energy might be recovered from small systems and stored efficiently is a complex one, despite the optimism shown in previous discussions of erasing. Indeed, current technology does not attempt to recover any energy from bits.

We now calculate the work done for our protocol (example 2.1). The particle’s initial potential energy is approximately *k*_B_*T*/2 on average, due to the equipartition theorem, and after the control is switched the average potential energy is *A*+*F*⋅*B* for a particle in the right well, since the particle is localized around *x*=*B*, and still *k*_B_*T*/2 for a particle in the left well. So, ignoring energy recovery at the end of the operation, the net work done for the erasure protocol is *W*=(*A*+*F*⋅*B*−*k*_B_*T*/2)/2. As justified analytically and numerically in the electronic supplementary material, §3.1, this approximation is accurate for the values of *A* and *F* that we consider, and we will use this as the form of work for the rest of the manuscript.

**Observation 2.2**

Work is an increasing function of well height *A* at fixed *F* and *γ*. This follows immediately from the expression of work *W*=(*A*+*F*⋅*B*−*k*_B_*T*/2)/2.

## 3. Friction-based trade-offs for reliability and erasing

We explore the behaviour of the reliability and erasing time scales as functions of the friction coefficient. We find that both these time scales are non-monotonic, roughly U-shaped functions of the friction coefficient. A high reliability time requirement is favoured by a very low or very high friction, whereas a low erasing time requirement is helped by the choice of a moderate value of friction. Since a bit designer would seek reliable bits (needing high or low friction) that can be erased fast (needing intermediate friction), this yields a friction-based trade-off between reliability and speed of erasure.

### (a) Reliability time

**Figure 3.**
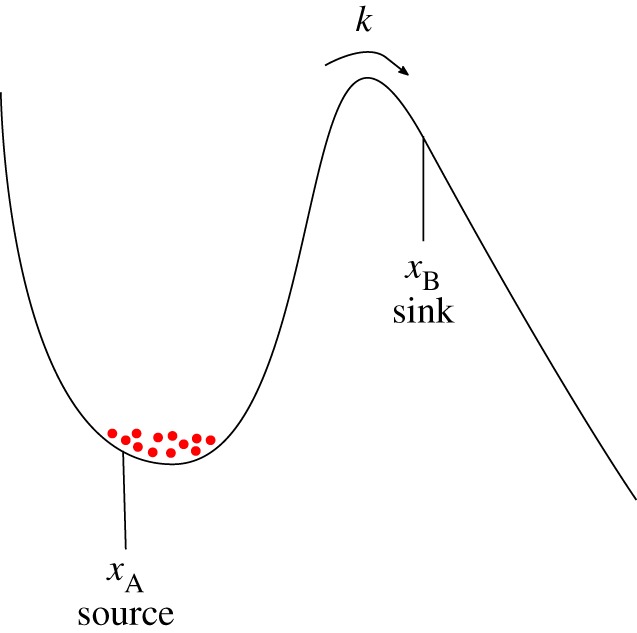
The escape of particles from a one-dimensional well. Kramers [40] considered a source of particles at the bottom of the well, and estimated the rate of escape to a sink on the far side of a barrier.

Our definition of reliability time (equation (2.4)) is very similar to the classic problem of escape rates from one-dimensional wells (figure 3), as applied in transition state theory to understand chemical reactions. In [40], Kramers found analytic expressions for the escape rate *k* from a well by calculating the flux of particles between a source on one side of the barrier (*x*_*A*_) and a sink on the other side (*x*_B_). Kramers’ expressions apply separately to the regimes of low friction, moderate to high friction and very high friction. Later the groups of Mel’nikov & Meshkov [41] and Pollak *et al.* [42] gave formulae that interpolate accurately over all values of friction (see the review in [43]). We will apply the result of Mel’nikov and Meshkov to estimate analytical forms of the escape rate for our bistable system

3.1 $$\begin{array}{rl} k & =\frac{\omega_{0}}{2\pi }\left[\sqrt{1+\frac{\gamma^{2}}{4\omega_{b}^{2}}}-\frac{\gamma }{2\omega_{b}}\right]g\;e^{-A/k_{B}T},\;\text{where}\\ \ln g & =\frac{1}{2\pi }\int_{0}^{\frac{\pi }{2}}\ln\left[1-\exp\left(\frac{-\gamma \;I(A)}{4k_{B}T\cos^{2}x}\right)\right]dx. \end{array}\}$$

Here, *ω*_*b*_ is the angular frequency at barrier height, *ω*_0_ is the angular frequency at the bottom of the well and *I*(*A*) is the action for barrier height *A* (see the electronic supplementary material, §5, for a detailed definition of these parameters and calculations for our system).

**Figure 4.**
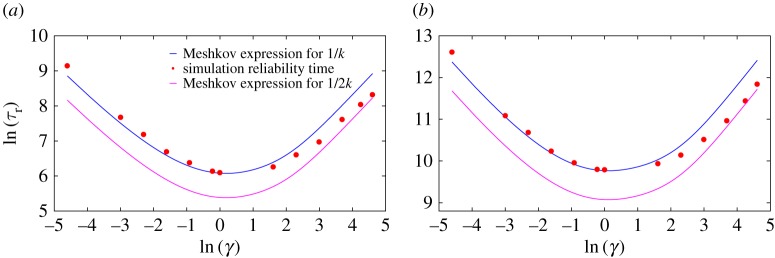
The reliability time *τ*_r_∝1/*γ* in the low-friction regime, *τ*_r_∝*γ* in the high-friction regime and is minimum at moderate friction. Simulation results are compared with the inverse of the escape rate from a single well (1/*k*) and (1/2*k*), as predicted by equation (3.1). Here, and elsewhere in the manuscript, error bars are omitted when comparable to data points. (*a*) *A*=6 and (*b*) *A*=10.

We plot the analytical prediction of 1/*k* given by equation (3.1) in figure 4 for two values of well height *A*, as a function of friction *γ*. This prediction is compared with average first passage time for particles to reach the top of the barrier from an initial Boltzmann distribution within a single well. The two quantities differ at large *γ* because Kramers’ definition does not treat a particle that crosses the barrier but then immediately crosses back as having ‘escaped’, whereas our definition of reliability in terms of a first passage time treats such particles as no longer being reliable. In the underdamped regime, immediate recrossings are rare and hence *τ*_r_ and 1/*k* coincide; in the overdamped regime, particles that reach the barrier top have a 50% chance of returning and so *τ*_r_=1/2*k*. As can be seen from figure 4, *τ*_r_ smoothly interpolates between 1/*k* and 1/2*k*, with the small numerical factor providing only a minor correction to the underlying physics of the analytical expression in equation (3.1).

The Mel’nikov–Meshkov expression predicts an almost-exponential scaling of 1/*k* with barrier height *A*, which is reproduced by *τ*_r_ and expected from the Arrhenius rate law [44]. Note that both 1/*k* and *τ*_r_ are non-monotonic in friction *γ*, with long reliability times in the underdamped and overdamped limits. This behaviour results from the need for particles to diffuse in both position and energy in order to reach the top of the barrier from an initial state thermalized within a single well. At high friction, particles rapidly sample different kinetic energies due to strong coupling with the environment, but move slowly in position space and hence take a long time to cross the barrier. At low friction, particles can move rapidly but their energy remains effectively constant over short time periods. They only cross the barrier when they have eventually gained enough total energy. Intermediate friction, when neither process is excessively slow, gives the shortest *τ*_r_. This behaviour is typical of equilibrating systems in which an initial out-of-equilibrium condition (particles are guaranteed to be on one side of the well and not on the other) relaxes towards an equilibrium state (particles on both sides of the barrier), and is thus insensitive to the details of our bit design.

A more detailed analysis of the dependence of the reliability time on various parameters, and indeed the functional form of the well, is possible. However, these details are not necessary for the conclusions we draw in the rest of this manuscript, and hence we omit them here.

### (b) Erasing time

As noted earlier, the erasing time is composed of two parts: the transport time defined in equation (2.10) and the mixing time defined in equation (2.11). We now present analytical estimates of these times and compare them with numerical solutions.

#### (i) Transport time

We can obtain an analytical estimate of the transport time in low- and high-friction limits by assuming that a particle starting at *x*=*B* moves deterministically under the influence of the potential slope and drag force.

1. *Low-friction regime*. The particle travels with a constant acceleration of $\frac{F}{m}$ and the time taken to travel a distance *B* is $\tau_{t}\approx \sqrt{2mB/F}$.
2. *High-friction regime*. In this regime, we assume that the net force on the particle (arising from the sum of drag and potential) is zero. The particle travels with a velocity of *F*/*mγ*, and the time taken to travel a distance *B* is *τ*_*t*_≈*mBγ*/*F*.

**Figure 5.**
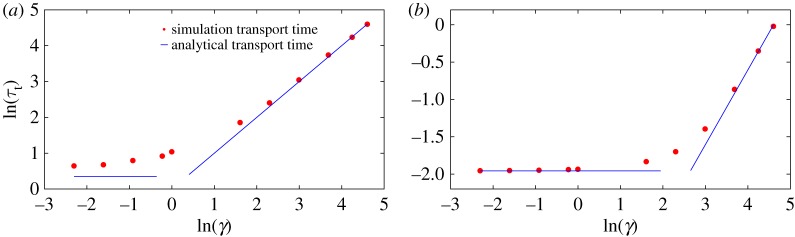
The transport time obtained from simulations approximates the analytical estimates of $\tau_{t}\approx \sqrt{2mB/F}$ in the low-friction regime, *τ*_*t*_≈*mBγ*/*F* (∝*γ*) in the high-friction regime. (*a*) *A*=10,*F*=1 and (*b*) *A*=10,*F*=100.

We thus expect the transport time to be constant in the underdamped regime and increase linearly with friction in the overdamped regime. Figure 5 illustrates that this scaling is observed in Langevin simulations, and that numerical values are in reasonable agreement with these crude estimates. The largest quantitative deviations occur at low force and low friction (e.g. *F*=1 in figure 5*a*), when the diffusion of the particle on the slope contributes significantly to *τ*_*t*_. This results in a simulation transport time larger than the analytical estimate.

#### (ii) Mixing time

Similar to the transport time, analytical estimates of the mixing time can be obtained in the limits of high and low friction.

1. *Low-friction regime*. For the purposes of an approximate calculation we treat the well ‘0’ as a harmonic oscillator. Deterministically, the energy of a harmonic oscillator decays exponentially in the underdamped regime. Therefore, we have *E*(*t*)=*E*_0_ *e*^−*γt*^, where *E*_0_ is the initial energy of the particle when it first reaches *x*=0 and *E*(*t*) is the energy of the particle at time *t*. In the underdamped regime, a particle starting at *B* arrives at position *x*=0 with energy *E*_0_≈*A*+*F*⋅*B*. Thus, solving for *E*(*τ*_*mix*_)=*A*−3*k*_B_*T*,
  3.2 $$\tau_{\mathrm{mix}}\approx \frac{1}{\gamma }\log\frac{A+F\cdot B}{A-3k_{B}T}.$$
2. *High-friction regime*. A sensible estimate of the behaviour can be obtained by explicitly modelling the diffusion of the particle near the barrier top. In the overdamped limit, the criterion of reaching a total energy of *E*(*τ*_*mix*_)=*A*−3*k*_B_*T* is equivalent to reaching a point *d* which has potential energy of *A*−3*k*_B_*T*, since momenta are sampled arbitrarily rapidly in this limit. To proceed, we consider the typical time required to reach an absorbing barrier at *d* starting from *x*=0, assuming a sufficiently large *F* that we can treat *x*=0 as a reflecting barrier. Starting from the overdamped stochastic differential equation
  3.3 $$m\gamma \;dx=-\partial_{x}U_{A,B}(x)\;dt+\sqrt{2m\gamma k_{B}T}\;dW,$$
  with generator $L=(k_{B}T/m\gamma )\;e^{U_{A,B}(x)/k_{B}T}\partial_{x}\;e^{-U_{A,B}(x)/k_{B}T}\partial_{x}$, we apply the standard methods outlined in Pavliotis [25], (7.1), p. 239, which leads to the following system of equations for the average mixing time *τ*_*mix*_(*x*) as a function of the initial position *x*:
  3.4 $$\begin{array}{rl} & \frac{k_{B}T}{m\gamma }\;e^{U_{A,B}(x)/k_{B}T}\partial_{x}\;e^{\frac{-U_{A,B}(x)}{k_{B}T}}\partial_{x}\tau_{\mathrm{mix}}(x)=-1,\;d<x\leq 0\\ \text{and}\; & \tau_{\mathrm{mix}}(x)=0,\;x=d. \end{array}\}$$
  We can solve equation (3.4) using appropriate limits to get
  3.5 $$\tau_{\mathrm{mix}}(x)=\frac{m\gamma }{k_{B}T}\int_{0}^{\sqrt{\frac{3k_{B}T}{2A}}B}\int_{0}^{q}e^{\frac{U(q)-U(r)}{k_{B}T}}\;dq\;dr,$$
  where we have approximated the potential near the barrier as an inverted harmonic oscillator to estimate $d=\sqrt{3k_{B}T/2A}B$. Repeating this approximation within the integral, we obtain
  3.6 $$\tau_{\mathrm{mix}}(x)\approx \frac{m\gamma }{k_{B}T}\int_{0}^{B\sqrt{\frac{3k_{B}T}{2A}}}\int_{0}^{q}e^{\frac{2A(r^{2}-q^{2})}{B^{2}k_{B}T}}\;dq\;dr\approx \frac{mB^{2}\gamma }{2\sqrt{2}A}.$$

**Figure 6.**
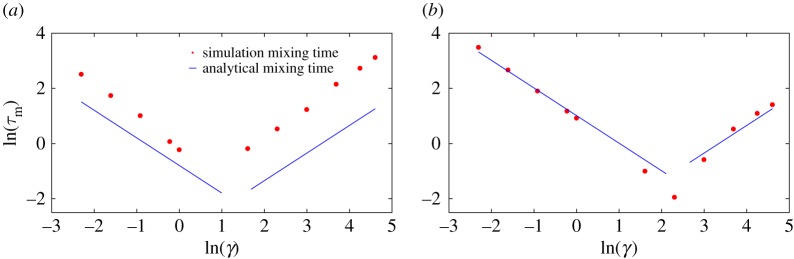
Evidence from simulation that the mixing time $\tau_{\mathrm{mix}}\approx (1/\gamma )\log\left((A+F\cdot B)/(A-3k_{B}T)\right)(\propto 1/\gamma )$ in the low-friction regime, $\tau_{\mathrm{mix}}\approx (mB^{2}\gamma /2\sqrt{2}A)(\propto \gamma )$ in the high-friction regime and is minimized at moderate friction. (*a*) *A*=10,*F*=1 and (*b*) *A*=10,*F*=100.

**Figure 7.**
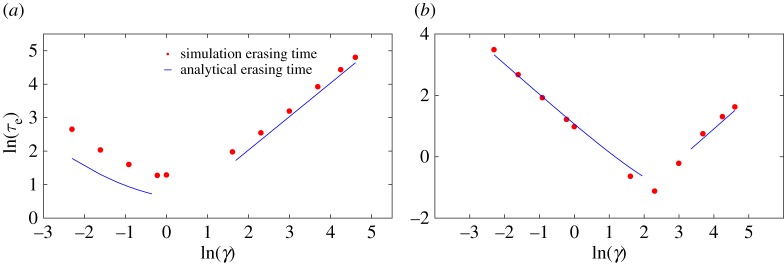
Evidence from simulation that the erasing time $\tau_{e}\approx \sqrt{2mB/F}+(1/\gamma )\log\left((A+F\cdot B)/(A-3k_{B}T)\right)$ in the low-friction regime, scaling as 1/*γ*, and $\tau_{e}\approx mB\gamma /F+mB^{2}\gamma /2\sqrt{2}A$ in the high-friction regime, scaling as *γ*. The erasing time is minimized at moderate friction. (*a*) *A*=10,*F*=1 and (*b*) *A*=10,*F*=100.

Equations (3.2) and (3.6) predict that the mixing time will scale as 1/*γ* in the low-friction limit and as *γ* in the high-friction limit. In the first case, mixing within the well is limited by the rate at which the particle can reduce its total energy, whereas, in the second, it is determined by the speed with which the particle can diffuse in position space to a configuration with lower potential energy. We plot simulation results for the mixing time, along with the analytic predictions, in figure 6, confirming this scaling and the resultant non-monotonicity. Quantitatively, simulation results deviate from the crude analytic predictions at low force (e.g. *F*=1 in figure 6*a*), when it is no longer reasonable to treat *x*=0 as either a reflecting barrier or a steep side of a harmonic well. Instead, excursions of the particle back onto the slope occupying the region *x*>0 lead to much larger mixing times. Nonetheless, the scaling and non-monotonicity in friction are preserved. Combining *τ*_trans_ and *τ*_mix_ gives *τ*_e_, plotted in figure 7. Analytically, the erasing time is given as:

1. *Low-friction regime*:
  3.7 $$\tau_{e}\approx \sqrt{\frac{2mB}{F}}+\frac{1}{\gamma }\log\frac{A+F\cdot B}{A-3k_{B}T}.$$
2. *High-friction regime*:
  3.8 $$\tau_{e}\approx \frac{mB\gamma }{F}+\frac{mB^{2}\gamma }{2\sqrt{2}A}.$$

**Figure 8.**
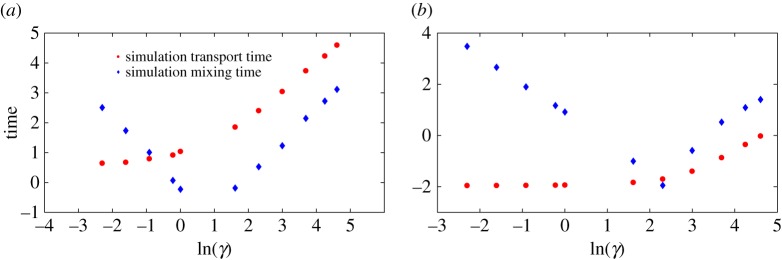
Comparison of transport and mixing times. Transport time dominates the mixing time for low force at high friction. (*a*) *A*=10,*F*=1 and (*b*) *A*=10,*F*=100.

Like reliability, erasing time is large in the underdamped and overdamped limits, and minimized at intermediate values of friction. The physical cause is the same as before; our erasing protocol involves setting the system into a non-equilibrium state, and waiting for the system to relax towards an equilibrium in the perturbed potential. This process requires the system to diffuse in energy space and also explore configuration space, and is therefore favoured by intermediate friction. Specifically, if the friction is too low, the particle oscillates and slowly loses energy to be confined within the desired well. If the friction is too high, both the transport and mixing times increase as the particle’s movement through space is so slow. The relative importance of these effects can be seen in figure 8. We note that the value of the damping *γ* that minimizes *τ*_e_ is quite sensitive to *F* (figure 8). Fundamentally, a larger *F* means the challenge of moving in position space is made easier, and a greater loss of energy is needed to reach equilibrium. Therefore, a higher friction coefficient is optimal. As with the reliability time, further analysis is possible but not necessary for the conclusions we wish to draw. Once again, the key point is the trade-off between high and low friction, which is not specific to our control. Indeed, it is likely to be quite generic since any protocol will necessarily push the system out of equilibrium, and will require particles to be typically confined within the target well before the control is removed.

Both erasing and reliability times exhibit a trade-off in friction, being minimized by intermediate values. This fact sets up a second trade-off between designing bits with extreme values of friction to optimize reliability, or moderate values of friction to optimize erasing. The consequences of this secondary trade-off will be explored in §4.

#### (iii) Additional dependencies of the erasing time

A larger value of *A* implies a steeper descent into the target left-hand well, making mixing faster. We therefore expect that the mixing time, and hence the erasing time, monotonically decreases with *A*.

**Observation 3.1**

**Figure 9.**
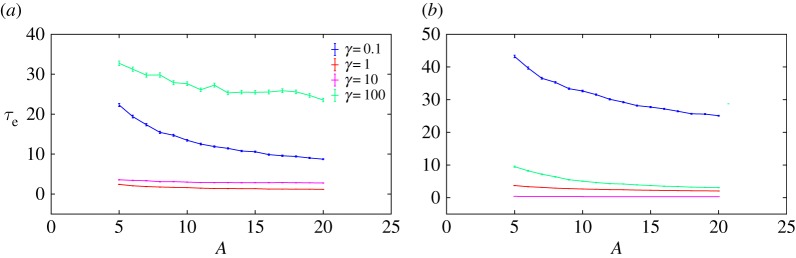
Evidence that the erasing time is a strictly decreasing function of well height across a range of *F* and *γ*. Other values of *F* and *γ* show similar behaviour. (*a*) *F*=5 and (*b*) *F*=100.

The erasing time is a strictly decreasing function of well height *A* at fixed *F*, *γ*. This can be seen from the analytic expressions of erasing time (equations (3.7) and (3.8)) backed up with numerical simulations (figure 9).

**Figure 10.**
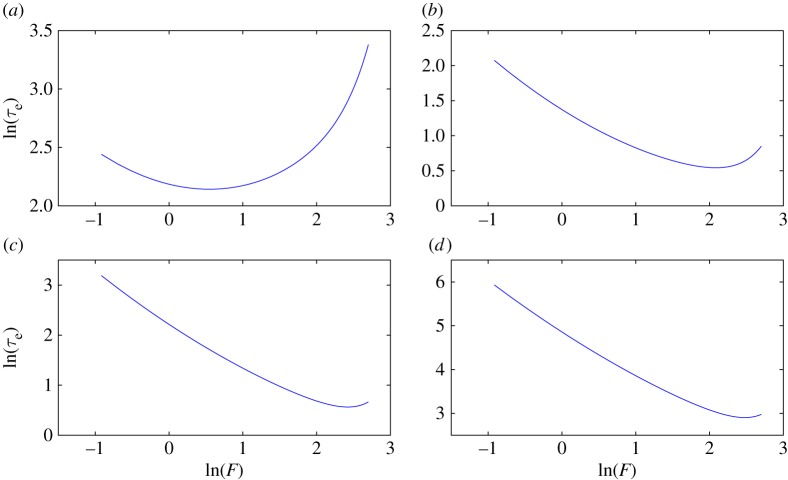
For a fixed value of *W* and *γ*, the erasing time is a non-monotonic function of *F* and is minimum at moderate *F*. This is illustrated at work *W*=20 for various values of *γ*. (*a*) *γ*=0.1, (*b*) *γ*=1, (*c*) *γ*=10 and (*d*) *γ*=100.

By contrast, erasing time shows a non-monotonic dependence on *F* at fixed *A*, *γ*. Applying too little force leads to slow transport, and does not effectively trap the particle within the target well. But applying too much force supplies the particle with too much energy, which must subsequently be lost during the mixing period. The fact that erasing time monotonically decreases with *A* at fixed *F* and *γ*, and shows a non-monotonic dependence on *F* at fixed *A* and *γ*, leads to a non-monotonic dependence of *τ*_e_ on *F* at fixed *W*=*A*+*F* and *γ*. We illustrate this non-monotonicity in figure 10, in which simple regression formulae have been fitted to the simulation data to enable interpolation at fixed *W* and *γ* (see electronic supplementary material, §4). As friction increases, the force required to provide the particle with excess energy increases, leading to minima at higher values of *F*.

We make the following observation, which will be used in the subsequent section.

**Observation 3.2**

We have found no evidence of multiple local minima of erasing time in a level set of work for our control family (see the electronic supplementary material, §6, for characterstic plots showing the minima of erasing time in a level set of work). Physically, this is unsurprising as the non-monotonicity in *τ*_e_ with *γ* and *F* mentioned above arises from fairly simple trade-offs, producing curves with single minima.

As with the reliability time, a more detailed analysis of the dependence of *τ*_e_ on other parameters, and even the shape of the control, is possible. However, these details are likely to be difficult to generalize, and are not necessary for the conclusions we draw in the subsequent sections.

## 4. Design of bits

We are now ready to study the question of how to design good bits. A design involves choosing parameters *A*,*F*,*γ* for a bit to satisfy requirement specifications in terms of speed of erasing and reliability, without expending more work than required. The most general formulation of our problem would require us to also allow the length scale *B*, the temperature *T* and the mass *m* to vary, as well as allowing arbitrary controls. Such a formulation would appear to make the problem even more challenging, so it seems prudent to restrict our first analysis to the variables *A*,*F* and *γ*. Our restricted analysis is not without value since the underlying technology in any given construction typically does not allow arbitrary variation. Our numerical analysis with example 2.1 will guide us in our assumptions and analyses, but our results will hold in greater generality. We will construct our proofs based on general assumptions, and subsequently explain how these assumptions are met by our control family.

We introduce the following terms.

1. The design of a bit is completely specified by the *design triple* (*A*,*F*,*γ*). *Design space* (DS) is the space of all design triples (*A*,*F*,*γ*).
2. A *requirement specification* is a tuple $(t_{r},t_{e})\in R_{>0}^{2}$ denoting the reliability and erasing time that we require of the bit. *Requirement space* (RS) is the space of all requirement specifications.
3. *Erasing time* $\tau_{e}:DS\to R_{>0}$ takes a design triple (*A*,*F*,*γ*) to the time required for erasing the corresponding bit under the control protocol specified by *F*. *Reliability time* $\tau_{r}:DS\to R_{>0}$ takes a design triple (*A*,*F*,*γ*) to the reliability time of the corresponding bit. Note that *τ*_r_ is constant as a function of *F* as it is a property of the dynamics in the absence of control.
4. *Work* $W:DS\to R_{>0}$ represents the expected work done by the control in erasing the corresponding bit. We will assume that *W* is constant as a function of *γ*, as is the case in example 2.1.
5. A design (*A*,*F*,*γ*) is *feasible* for a requirement (*t*_r_,*t*_e_) iff both *τ*_r_(*A*,*F*,*γ*)≥*t*_r_ and *τ*_e_(*A*,*F*,*γ*)≤*t*_e_. A (*t*_r_,*t*_e_)-feasible design (*A*,*F*,*γ*) is (*t*_r_,*t*_e_)-*optimal* iff the work *W*(*A*,*F*,*γ*) is minimum among all (*t*_r_,*t*_e_)-feasible designs.
6. Inspired by the observation that non-trivial minima of erasing time at fixed work exist for our family of protocols (§3b(iii)), we define the notion of *trapped* bits. A design (*A*,*F*,*γ*) is *trapped* iff for all designs (*A*′,*F*′,*γ*′) with *W*(*A*,*F*,*γ*)=*W*(*A*′,*F*′,*γ*′) the erasing time *τ*_e_(*A*,*F*,*γ*)≤*τ*_e_(*A*′,*F*′,*γ*′). A design (*A*,*F*,*γ*) is *uniquely trapped* iff for all designs (*A*′,*F*′,*γ*′) with *W*(*A*,*F*,*γ*)=*W*(*A*′,*F*′,*γ*′) the erasing time *τ*_e_(*A*,*F*,*γ*)≤*τ*_e_(*A*′,*F*′,*γ*′) with equality iff (*A*,*F*,*γ*)=(*A*′,*F*′,*γ*′). A design (*A*,*F*,*γ*) is *locally trapped* iff there exists a neighbourhood of (*A*,*F*,*γ*) consisting of bits (*A*′,*F*′,*γ*′) with *W*(*A*,*F*,*γ*)=*W*(*A*′,*F*′,*γ*′) such that the erasing time *τ*_e_(*A*,*F*,*γ*)≤*τ*_e_(*A*′,*F*′,*γ*′). More informally, a trapped design has the lowest erasing time within a level set of work; a trapped design is unique if it is the *only* design within that level set of work to have the minimal erasing time; and a locally trapped design has the minimal erasing time within a local neighbourhood of designs of equal work.
7. A requirement specification (*t*_r_,*t*_e_) is *unsaturated* iff there exists a (*t*_r_,*t*_e_)-optimal design (*A*,*F*,*γ*) such that either *τ*_r_(*A*,*F*,*γ*)>*t*_r_ or *τ*_e_(*A*,*F*,*γ*)<*t*_e_. A feasible requirement specification that is not unsaturated is called *saturated*.

Throughout this section, we will assume that *τ*_e_,*τ*_r_ and *W* are continuous functions.

We will state the main results related to the properties of the optimal design leaving the detailed proofs to the electronic supplementary material. We first claim that an optimal design always saturates the bound on the erasing time constraint. Further, if the optimal bit is not locally trapped, then it also saturates the bound on the reliability time constraint.

**Claim 4.1 saturation of time scales**

Let us assume that it is possible to locally decrease work at a fixed reliability time. (This is generally possible since one can perturb the control parameters to reduce work; but reliability time does not depend on the control parameters.) Fix requirement specifications $(t_{r},t_{e})\in RS$. Suppose (*A*,*F*,*γ*) is a (*t*_r_,*t*_e_)-optimal design. Then

1. *τ*_e_(*A*,*F*,*γ*)=*t*_e_.
2. If the design (*A*,*F*,*γ*) is not locally trapped, then *τ*_r_(*A*,*F*,*γ*)=*t*_r_.

**Proof.**

See the electronic supplementary material, §7. ■

The next claim provides insight into the geometry of optimal designs. In particular, it states that under mild assumptions the requirement space is divided into two regions by a boundary given by the reliability and erasing times of trapped designs. Requirements with *t*_r_<*t*^′^_*r*_ and $t_{e}=t_{e}^{\prime }$, where $\left(t_{r}^{\prime },t_{e}^{\prime }\right)$ is a requirement on the dividing line, are unsaturated, while other requirement specifications are saturated.

**Claim 4.2 saturated and unsaturated requirements**

Assume that the erasing time of trapped designs is a strictly decreasing function of the work (see the electronic supplementary material, observation 7.1, for a justification), and that as before it is always possible to decrease work at a fixed reliability time. Let (A*,F*,γ*) be a trapped design such that *τ*_e_(A*,F*,γ*)=*t*_e_.

1. If *t*_r_≤*τ*_r_(A*,F*,γ*) then (A*,F*,γ*) is (*t*_r_,*t*_e_)-optimal.
2. If *t*_r_<*τ*_r_(A*,F*,γ*) then (*t*_r_,*t*_e_) is unsaturated.
3. Make the additional assumption that locally trapped designs are uniquely trapped (as noted for our family of protocols (example 2.1) in observation 3.2).
  If *t*_r_≥*τ*_r_(A*,F*,γ*), then (*t*_r_,*t*_e_) is saturated.

**Proof.**

See the electronic supplementary material, §7. ■

The claims about saturation/unsaturation of time scales can also be proved using Karush–Kuhn–Tucker (KKT) conditions (see the electronic supplementary material, §7), a standard tool from optimization theory.

**Figure 11.**
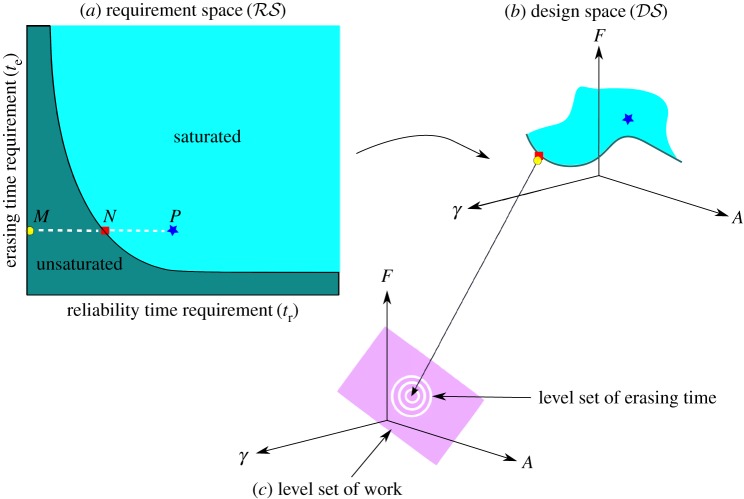
An illustration of the mapping of requirement specifications to optimal designs. The design space (DS) is divided by a curve corresponding to the reliability and erasing times of trapped designs. Points *M*,*N* in (*a*) requirement space (RS) having the same erasing time requirement get mapped to the same optimal bit in (*b*) design space : a trapped design with *τ*_r_ and *τ*_e_ equal to the requirements at *N*. The requirement specifications represented by points like *P* having the same *t*_e_ but greater *t*_r_ than *N* are mapped to distinct points in design space. (*c*) A representation of a level set of work *W* within the design space, illustrating that the optimal designs to which unsaturated requirements are mapped minimize the erasing time among all designs requiring the same work.

A more intuitive picture of the results can be understood from figure 11. In this figure, we illustrate how finding an optimal design subject to a specification maps a point in the requirement space to a point in the design space. For a trapped design (A*,F*,γ*), requirements with *t*_r_<*τ*_r_(A*,F*,γ*) and *t*_e_=*τ*_e_(A*,F*,γ*) are unsaturated and get mapped to the same design (A*,F*,γ*) (claims 4.2(2) and 4.2(1)). If the design (A*,F*,γ*) is uniquely trapped, then requirements with *t*_r_≥*τ*_r_(A*,F*,γ*) and *t*_e_=*τ*_e_(A*,F*,γ*) are saturated (claim 4.2(3)).

**Figure 12.**
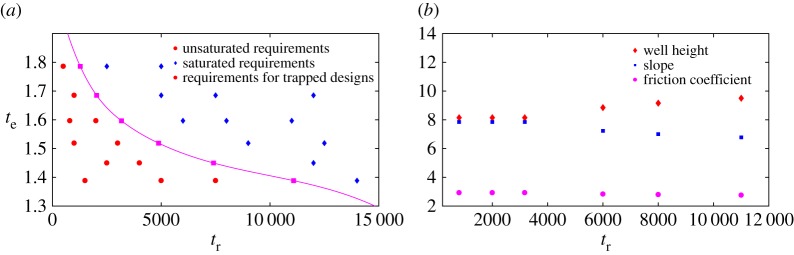
Illustration of the division of requirement space (RS) into saturated and unsaturated regions by requirements that correspond to trapped designs. (*a*) Squares show requirements (*t*_e_,*t*_r_) that are saturated by trapped designs for the family of protocols we consider. Numerical optimization shows that requirements to the left of the locus defined by these points are unsaturated (circles), whereas requirements to the right are saturated (diamonds). (*b*) A plot of the optimal designs for points from (*a*) at *t*_e_=1.5967. It is clear that for requirements *t*_r_≤3173, lying to the left of the trapped-design locus in (*a*), optimal design parameters are identical, whereas they are distinct for *t*_r_>3173.

Figure 12 illustrates these results for our example family of controls (example 2.1). As discussed in the electronic supplementary material, §4, we have implemented simple regression to fit the functions *τ*_e_(.) and *τ*_r_(.) to our simulation results. We then identified trapped designs using numerical minimization, plotting the requirement specifications saturated by these designs. For each trapped bit (A*,F*,γ*), we randomly selected requirements with *t*_e_=*τ*_e_(A*,F*,γ*), but with *t*_r_ either greater than, equal to or less than *τ*_r_(A*,F*,γ*), and used numerical optimization techniques to search for the optimal designs. The results support our analysis; requirements with *t*_r_<*τ*_r_(A*,F*,γ*) are unsaturated, and those with *t*_r_≥*τ*_r_(A*,F*,γ*) are saturated. Furthermore, as we show in figure 12*b*, unsaturated requirements at fixed *t*_e_ all map to the same trapped design.

### (a) Optimal friction for simple controls

In §3, we demonstrated that both reliability and erasing times are non-monotonic in friction, with short erasing times favoured by moderate values of friction, and long reliability times favoured by extreme values. In what follows, we give a precise quantification of the resultant trade-off in finding the friction of an optimal bit. The analysis is significantly simplified for our family of controls, in which work is independent of the friction coefficient.

Let us introduce the following terms. Fix *A* and *F*. Then,

1. *γ*^*e*^_*crit*_ is the friction coefficient that minimizes the erasing time as a function of friction coefficient *γ* at fixed *A* and *F*, i.e. for all $\gamma^{\prime }\in R_{>0}$, we have
  4.1 $$\tau_{e}(A,F,\gamma_{\mathrm{crit}}^{e})\leq \tau_{e}(A,F,\gamma^{\prime }).$$
  We call the design $\left(A,F,\gamma_{\mathrm{crit}}^{e}\right)$ *critically damped*.
2. *γ*^*r*^_*crit*_ is the friction coefficient that minimizes the reliability time as a function of friction coefficient *γ* at fixed *A* and *F*, i.e. for all $\gamma^{\prime }\in R_{>0}$, we have
  4.2 $$\tau_{r}(A,F,\gamma_{\mathrm{crit}}^{r})\leq \tau_{r}(A,F,\gamma^{\prime }).$$

**Figure 13.**
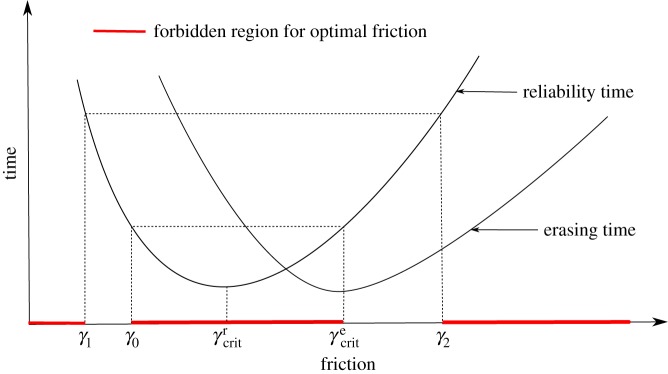
Regions of friction space can be eliminated from the search for optimal bits for our class of controls. As a result, the optimal friction either is critical damping or it lies somewhere within the two regions of moderate friction. Illustrative curves of *τ*_e_ and *τ*_r_ at fixed *A*,*F* indicate these regions.

It is easy to note that trapped bits are also critically damped. In figure 13, we show illustrative curves of the erasing and reliability times as a function of friction coefficient *γ* at fixed *A*,*F*. These curves have single minima at *γ*^*e*^_*crit*_ and *γ*^*r*^_*crit*_, respectively. Also shown on these graphs are regions of friction space that can be eliminated from consideration for optimal bits. To eliminate extreme values of friction, we note that the design must have a minimal finite *A* to be a well-defined two-state system in the resting state. For our bit, it is *A*_*min*_≈3. In the next claim, we precisely describe which regions of friction can be eliminated.

**Claim 4.3 forbidden regions for optimal friction**

Assume that both *τ*_e_ and *τ*_r_ have a single, well-defined minimum and tend to infinity as *γ* tends to zero or infinity. Let (*A*,*F*,*γ*) be a (*t*_r_,*t*_e_)-optimal design (see figure 13 for notational convenience).

1. Let *γ*_0_ be such that $\tau_{r}(A,F,\gamma_{0})=\tau_{r}(A,F,\gamma_{\mathrm{crit}}^{e})$.
  - If $\gamma_{\mathrm{crit}}^{e}>\gamma_{\mathrm{crit}}^{r}$, then *γ*∉(*γ*_0_,*γ*^*e*^_*crit*_).
  - If $\gamma_{\mathrm{crit}}^{e}<\gamma_{\mathrm{crit}}^{r}$, then $\gamma \notin (\gamma_{\mathrm{crit}}^{e},\gamma_{0})$.
  That is, the friction of the optimal bit does not reside in the central red region in figure 13.
2. Let *A*_*min*_ be the minimum height for a bit to be meaningfully bistable and let *γ*_1_<*γ*_2_ be such that *τ*_r_(*A*_*min*_,*F*,*γ*_1_)=*τ*_r_(*A*_*min*_,*F*,*γ*_2_)=*t*_r_. If (*A*,*F*,*γ*) is not locally trapped, then $\gamma \notin (0,\gamma_{1})\cup (\gamma_{2},\infty )$.
  That is, the friction of the optimal bit does not arise from the extreme red regions in figure 13.

**Proof.**

1. We prove it for the case when $\gamma_{\mathrm{crit}}^{e}>\gamma_{\mathrm{crit}}^{r}$; the other case proceeds in identical fashion. For contradiction, assume that *γ*∈(*γ*_0_,*γ*^*e*^_*crit*_). Then, due to the single minima in both *τ*_e_ and *τ*_r_, and the fact that *τ*_r_ tends to infinity as *γ* tends to zero or infinity, there exists a design (*A*,*F*,*γ*^′^) with *γ*^′^>*γ*_0_ and *τ*_r_(*A*,*F*,*γ*^′^)=*τ*_r_(*A*,*F*,*γ*)≥*t*_r_, but *τ*_e_(*A*,*F*,*γ*^′^)<*τ*_e_(*A*,*F*,*γ*)≤*t*_e_. The design (*A*,*F*,*γ*^′^) is (*t*_r_,*t*_e_)-optimal since it is (*t*_r_,*t*_e_)-feasible and has *W*(*A*,*F*,*γ*^′^)=*W*(*A*,*F*,*γ*), contradicting lemma 4.1(1) that the optimal bit saturates the bound on the erasing time constraint.
2. For contradiction, suppose that *γ*<*γ*_1_ or *γ*>*γ*_2_. Then since *A*≥*A*_*min*_ and the reliability time increases with well height and more extreme values of *γ*, either *τ*_r_(*A*,*F*,*γ*)≥*τ*_r_(*A*_*min*_,*F*,*γ*)>*τ*_r_(*A*_*min*_,*F*,*γ*_1_)=*t*_r_ or *τ*_r_(*A*,*F*,*γ*)≥*τ*_r_(*A*_*min*_,*F*,*γ*)>*τ*_r_(*A*_*min*_,*F*,*γ*_2_)=*t*_r_, contradicting claim 4.1(2) that an optimal design that is not locally trapped saturates the bound on the reliability time constraint. ■

**Figure 14.**
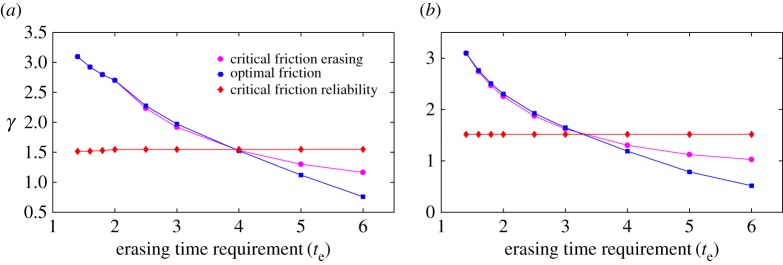
Optimal friction either is critical damping or lies within a small region adjacent to critical damping, for our family of controls. We plot friction for optimal designs (*A*,*F*,*γ*) against erasing time requirements (*t*_e_) for a fixed value of reliability time requirement (*t*_r_), alongside *γ*^*e*^_*crit*_ and *γ*^*r*^_*crit*_. Note that *A* and *F* are not fixed, but determined by the optimization procedure alongside the optimal friction for each requirement (*t*_r_,*t*_e_). The data were obtained from numerical optimization and minimization based on regression fits to simulation data. (*a*) Reliability time requirement (*t*_r_)=500 and (*b*) reliability time requirement (*t*_r_)=10 000.

For clarity, let us assume initially that $\gamma_{\mathrm{crit}}^{e}>\gamma_{\mathrm{crit}}^{r}$ (equivalent arguments hold for the alternative). We see that optimal designs either reside at *γ*^*e*^_*crit*_ or lie within two regions at moderate friction, as illustrated in figure 13. Interestingly, one region is adjacent to *γ*^*e*^_*crit*_, whereas the other is not. It is not easy to see how designs in one region (*γ*_1_≤*γ*≤*γ*_0_) as in figure 13 can outperform those in the other region (*γ*^*e*^_*crit*_<*γ*≤*γ*_2_). Indeed, when we performed numerical optimization on the regression-based fits to our simulation data, we only observed optimal bits that are either critically damped or lie in the allowed region adjacent to critical damping. This is illustrated in figure 14, where we plot the optimal friction as a function of erasing time requirement at fixed reliability time requirement, for two values of reliability time requirements. We also plot *γ*^*e*^_*crit*_ and *γ*^*r*^_*crit*_ for comparison. At low erasing time requirements, designs reside at *γ*^*e*^_*crit*_. At slightly higher erasing time requirements, the designs become saturated and the optimal friction lies adjacent to *γ*^*e*^_*crit*_ in the region *γ*^*e*^_*crit*_<*γ*≤*γ*_2_. Eventually, *γ*^*e*^_*crit*_ crosses *γ*^*r*^_*crit*_. At the crossing point, we have $\gamma =\gamma_{\mathrm{crit}}^{e}=\gamma_{\mathrm{crit}}^{r}$. At higher values of erasing time requirements, *γ* still occupies the region adjacent to *γ*^*e*^_*crit*_, which is now $\gamma_{1}\leq \gamma \leq \gamma_{\mathrm{crit}}^{e}<\gamma_{\mathrm{crit}}^{r}$.

## 5. Conclusion

We have explored the question of the design of optimal bits. Previously, authors have focused on designing optimal protocols that minimize work input when implementing a finite-time operation on a given system [8,45–48]. Our approach differs in considering that bits need to have two distinct functionalities: retain data for long periods of time and allow rapid switching or erasing. Moreover, we consider optimizing over system parameters such as the intrinsic friction as well as the external control. Our fundamental observation is that friction plays a non-trivial role in the design of bits. Both switching/erasing and the eventual degradation of data involve relaxation towards equilibrium from a non-equilibrium distribution. This process is fastest at intermediate values of the friction, but slow in the overdamped and underdamped regimes. The best bit designs have high reliability times and low switching/erasing times, which implies an inherent trade-off in bit design between extreme values of friction that favour high reliability, and moderate values of friction that favour rapid switching or erasing.

We have explored the consequences of the biphasic role of friction for a simple class of controls. The existence of non-trivial minima of erasing time in the level set of work leads to the generation of trapped designs. These designs are optimal for reliability requirements smaller than their own reliability time leading to unsaturated requirements. The result of the trade-off between extreme values of friction that maximize reliability time and moderate values of friction that minimize erasing times is that optimal designs are either critically damped or occupy a region of moderate friction close to critical damping.

Our work opens up a new perspective on the design of efficient computational devices showing that: *the best designs are likely to be neither underdamped nor overdamped*. This observation is particularly important as some authors have considered friction to be inherently problematic for computation [20–23]. Equally, the role of friction is suppressed when bits are modelled as discrete two-state systems [2,9,49], since this approximation assumes rapid equilibration within the discrete states.

We have only considered a simple family of controls to motivate our analysis and illustrate our findings. This family is not optimal—it was chosen for its simplicity and ease of analysis. Moreover, there is some arbitrariness in the definition of both the erasing and reliability times. As such, the numerical details of the results obtained are not very important. We are not claiming to have derived numerical corrections to the minimal cost of erasing a bit, for example, or the specific work costs (substantially larger than $k_{B}T\ln2$) which are not that informative. Rather, it is the qualitative results, which hold for a much broader class of controls, that are important. The non-monotonic role of friction in both the erasing and reliability time scales is a generic physical phenomenon that extends beyond the details of our implementation, and implies a competition between the goals of fast manipulation and long reliability times. Relatively weak assumptions—that it is always possible to decrease work at fixed reliability time and that the minimal erasing time decreases with increased work—imply that erasing time requirements are always saturated by optimal bits and that trapped designs lead to unsaturated reliability time requirements, respectively. Other results rely more on the simplicity of the control family: the existence of only one local minimum of erasing time at fixed work simplifies the question of whether a requirement specification is saturated. The fact that work is independent of friction simplifies the task of eliminating certain values of friction as suboptimal.

Explicit exploration of a broader class of controls, including those with more complex variation over time, and varying parameters such as particle mass and distance between wells, are possible directions for future work. It is not immediately clear whether minima in erasing time at fixed work cost will become more or less prominent features of the optimization landscape when the complexity of the system is increased, for example. In particular, raising or lowering the barrier between metastable states is a common idea [8,9,28,29]. Lowering the barrier during erasing potentially allows for faster erasing at fixed reliability time and lower work cost. If said barriers could be raised and lowered arbitrarily far and quickly, it may be possible to circumvent any conflict between high reliability and low erasure time. However, real physical systems are not generally this flexible. Indeed, in order to apply a complex time-dependent control to a small colloid, experimenters typically use optical feedback traps [28,29], which are not true potentials and rely on the continuous input of energy to apply forces and perform feedback control. For true physical protocols that permit finite raising and lowering of barriers between metastable states, we expect that our findings would still apply to a family of protocols with optimal barrier manipulation. An alternative direction would be to consider similar effects in systems with inherently quantum mechanical behaviour.

## Supplementary Material

Supplementary Information

## Data accessibility

The codes and computational data for this manuscript can be downloaded from https://www.imperial.ac.uk/principles-of-biomolecular-systems/contact-obtain-code-and-data/.

## References

[1] Szilard L 1964 On the decrease of entropy in a thermodynamic system by the intervention of intelligent beings. *Behav. Sci.* 9, 301–310. (doi:10.1002/bs.3830090402) 5888785 10.1002/bs.3830090402

[2] Landauer R 1961 Irreversibility and heat generation in the computing process. *IBM J. Res. Dev.* 5, 183–191. (doi:10.1147/rd.53.0183)

[3] Bennett CH 1982 The thermodynamics of computation—a review. *Int. J. Theor. Phys.* 21, 905–940. (doi:10.1007/BF02084158)

[4] Bennett CH 1988 Notes on the history of reversible computation. *IBM J. Res. Dev.* 32, 16–23. (doi:10.1147/rd.321.0016)

[5] Bennett CH 2003 Notes on Landauer’s principle, reversible computation, and Maxwell’s demon. *Stud. History Philos. Mod. Phys.* 34, 501–510. (doi:10.1016/S1355-2198(03)00039-X)

[6] Frank MP 2002 The physical limits of computing. *Comput. Sci. Eng.* 4, 16–26. (doi:10.1109/5992.998637)

[7] Pop E 2010 Energy dissipation and transport in nanoscale devices. *Nano Res.* 3, 147–169. (doi:10.1007/s12274-010-1019-z)

[8] Zulkowski PR, DeWeese MR 2014 Optimal finite-time erasure of a classical bit. *Phys. Rev. E* 89, 052140 (doi:10.1103/PhysRevE.89.052140) 10.1103/PhysRevE.89.052140 25353772

[9] Ouldridge TE, Govern CC, Wolde PR 2017 The thermodynamics of computational copying in biochemical systems. *Phys. Rev. X* 7, 021004 (doi:10.1103/PhysRevX.7.021004)

[10] Von Neumann J, Burks A 1966 Theory of self-reproducing automata. *IEEE Trans. Neural Netw.* 5, 3–14.

[11] Sarpeshkar R 2010 *Ultra low power bioelectronics: fundamentals, biomedical applications, and bio-inspired systems*. Cambridge, UK: Cambridge University Press.

[12] Sarpeshkar R 1998 Analog versus digital: extrapolating from electronics to neurobiology. *Neural Comput.* 10, 1601–1638. (doi:10.1162/089976698300017052) 9744889 10.1162/089976698300017052

[13] Rapoport BI, Kedzierski JT, Sarpeshkar R 2012 A glucose fuel cell for implantable brain–machine interfaces. *PLoS ONE* 7, e38436 (doi:10.1371/journal.pone.0038436) 22719888 10.1371/journal.pone.0038436 PMC3373597

[14] Tu Y 2008 The nonequilibrium mechanism for ultrasensitivity in a biological switch: sensing by Maxwell’s demons. *Proc. Natl Acad. Sci. USA* 105, 11 737–11 741. (doi:10.1073/pnas.0804641105) 18687900 10.1073/pnas.0804641105 PMC2575293

[15] Lan G, Sartori P, Neumann S, Sourjik V, Tu Y 2012 The energy-speed-accuracy trade-off in sensory adaptation. *Nat. Phys.* 8, 422–428. (doi:10.1038/nphys2276) 22737175 10.1038/nphys2276 PMC3378065

[16] Govern CC, ten Wolde PR 2014 Optimal resource allocation in cellular sensing systems. *Proc. Natl Acad. Sci. USA* 111, 17486–17491. (doi:10.1073/pnas.1411524111) 25422473 10.1073/pnas.1411524111 PMC4267345

[17] Ouldridge TE, tenWolde PR 2017 Fundamental costs in the production and destruction of persistent polymer copies. *Phys. Rev. Lett.* 118, 158103 (doi:10.1103/PhysRevLett.118.158103) 28452507 10.1103/PhysRevLett.118.158103

[18] Bo S, Giudice MD, Celani A 2015 Thermodynamic limits to information harvesting by sensory systems. *J. Stat. Mech. Theory Exp.* 2015, P01014 (doi:10.1088/1742-5468/2015/01/P01014)

[19] Barato AC, Hartich D, Seifert U 2014 Efficiency of cellular information processing. *New J. Phys.* 16, 103024 (doi:10.1088/1367-2630/16/10/103024)

[20] Anacker W 1980 Josephson computer technology: an IBM research project. *IBM J. Res. Dev.* 24, 107–112. (doi:10.1147/rd.242.0107)

[21] Büttiker M, Harris EP, Landauer R 1983 Thermal activation in extremely underdamped Josephson-junction circuits. *Phys. Rev. B* 28, 1268–1275. (doi:10.1103/PhysRevB.28.1268)

[22] Klein M, Mukherjee A 1982 Thermal noise induced switching of Josephson logic devices. *Appl. Phys. Lett.* 40, 744–747. (doi:10.1063/1.93213)

[23] Likharev KK 1982 Classical and quantum limitations on energy consumption in computation. *Int. J. Theor. Phys.* 21, 311–326. (doi:10.1007/BF01857733)

[24] Kloeden PE, Platen E 1992 Higher-order implicit strong numerical schemes for stochastic differential equations. *J. Stat. Phys.* 66, 283–314. (doi:10.1007/BF01060070)

[25] Pavliotis GA 2014 *Stochastic processes and applications*, vol. 60 Berlin, Germany: Springer.

[26] Mattingly JC, Stuart AM 2002 Geometric ergodicity of some hypo-elliptic diffusions for particle motions. *Markov Process. Relat. Fields* 8, 199–214.

[27] Gopalkrishnan M 2013 The hot bit I: the Szilard-Landauer correspondence. (http://arxiv.org/abs/1311.3533)

[28] Bérut A, Arakelyan A, Petrosyan A, Ciliberto S, Dillenschneider R, Lutz E 2012 Experimental verification of Landauer’s principle linking information and thermodynamics. *Nature* 483, 187–189. (doi:10.1038/nature10872) 22398556 10.1038/nature10872

[29] Jun Y, Gavrilov M, Bechhoefer J 2014 High-precision test of Landauer’s principle in a Feedback trap. *Phys. Rev. Lett.* 113, 190601 (doi:10.1103/PhysRevLett.113.190601) 25415891 10.1103/PhysRevLett.113.190601

[30] Sagawa T, Ueda M 2012 Nonequilibrium thermodynamics of feedback control. *Phys. Rev. E* 85, 021104 (doi:10.1103/PhysRevE.85.021104) 10.1103/PhysRevE.85.021104 22463150

[31] Sagawa T, Ueda M 2010 Generalized Jarzynski equality under nonequilibrium feedback control. *Phys. Rev. Lett.* 104, 090602 (doi:10.1103/PhysRevLett.104.090602) 20366975 10.1103/PhysRevLett.104.090602

[32] Ponmurugan M 2010 Generalized detailed fluctuation theorem under nonequilibrium feedback control. *Phy. Rev. E* 82, 031129 (doi:10.1103/PhysRevE.82.031129) 10.1103/PhysRevE.82.031129 21230047

[33] Abreu D, Seifert U 2012 Thermodynamics of genuine nonequilibrium states under feedback control. *Phys. Rev. Lett.* 108, 030601 (doi:10.1103/PhysRevLett.108.030601) 22400724 10.1103/PhysRevLett.108.030601

[34] Horowitz JM, Vaikuntanathan S 2010 Nonequilibrium detailed fluctuation theorem for repeated discrete feedback. *Phys. Rev. E* 82, 061120 (doi:10.1103/PhysRevE.82.061120) 10.1103/PhysRevE.82.061120 21230657

[35] Sourabh L, Shubhashis R, Jayannavar AM 2012 Fluctuation theorems in the presence of information gain and feedback. *J. Phys. A Math. Theor.* 45, 065002 (doi:10.1088/1751-8113/45/6/065002)

[36] Vega JL, Guantes R, Miret-Artes S 2002 Mean first passage time and the Kramers turnover theory in activated atom-surface diffusion. *Phys. Chem. Chem. Phys.* 4, 4985–4991. (doi:10.1039/B204462E)

[37] Ruslan LD, Richard H, Tretyakov MV 2009 Langevin thermostat for rigid body dynamics. *J. Chem. Phys.* 130, 234101 (doi:10.1063/1.3149788) 19548705 10.1063/1.3149788

[38] Sekimoto K 1997 Kinetic characterization of heat bath and the energetics of thermal ratchet models. *J. Phys. Soc. Jpn* 66, 1234–1237. (doi:10.1143/JPSJ.66.1234)

[39] Sekimoto K 1998 Langevin equation and thermodynamics. *Prog. Theor. Phys. Suppl.* 130, 17–27. (doi:10.1143/PTPS.130.17)

[40] Kramers HA 1940 Brownian motion in a field of force and the diffusion model of chemical reactions. *Physica* 7, 284–304. (doi:10.1016/S0031-8914(40)90098-2)

[41] Mel’nikov VI, Meshkov SV 1986 Theory of activated rate processes: exact solution of the Kramers problem. *J. Chem. Phys.* 85, 1018–1027. (doi:10.1063/1.451844)

[42] Pollak E, Grabert H, Hänggi P 1989 Theory of activated rate processes for arbitrary frequency dependent friction: solution of the turnover problem. *J. Chem. Phys.* 91, 4073–4087. (doi:10.1063/1.456837)

[43] Hänggi P, Talkner P, Borkovec M 1990 Reaction-rate theory: fifty years after Kramers. *Rev. Mod. Phys.* 62, 251–341. (doi:10.1103/RevModPhys.62.251)

[44] Arrhenius S 1889 *Über die Dissociationswärme und den Einfluss der Temperatur auf den Dissociationsgrad der Elektrolyte*. Leipzig, Germany: Wilhelm Engelmann.

[45] Schmiedl T, Seifert U 2007 Optimal finite-time processes in stochastic thermodynamics. *Phys. Rev. Lett.* 98, 108301 (doi:10.1103/PhysRevLett.98.108301) 17358574 10.1103/PhysRevLett.98.108301

[46] Then H, Engel A 2008 Computing the optimal protocol for finite-time processes in stochastic thermodynamics. *Phys. Rev. E* 77, 041105 (doi:10.1103/PhysRevE.77.041105) 10.1103/PhysRevE.77.041105 18517576

[47] Aurell E, Mejía-Monasterio C, Muratore-Ginanneschi P 2011 Optimal protocols and optimal transport in stochastic thermodynamics. *Phys. Rev. Lett.* 106, 250601 (doi:10.1103/PhysRevLett.106.250601) 21770620 10.1103/PhysRevLett.106.250601

[48] Gingrich TR, Rotskoff GM, Crooks GE, Geissler PL 2016 Near-optimal protocols in complex nonequilibrium transformations. *Proc. Natl Acad. Sci. USA* 113, 10 263–10 268. (doi:10.1073/pnas.1606273113) 27573816 10.1073/pnas.1606273113 PMC5027427

[49] Gopalkrishnan M 2016 A cost/speed/reliability tradeoff to erasing. *Entropy* 18, 165 (doi:10.3390/e18050165)

