# Supplementary material for: Designing the optimal bit: balancing energetic cost, speed and reliability
Source: Proc Math Phys Eng Sci. 2017 Aug 23;473(2204):20170117. doi: 10.1098/rspa.2017.0117 (PMC5582178; doi:10.1098/rspa.2017.0117)
Supplement: Supplementary Information [file rspa20170117supp1.zip › Supplementary/supplementary_information.pdf]

SUPPLEMENTARY INFORMATION FOR Designing the Optimal Bit: Balancing Energetic Cost, Speed and Reliability

Abhishek Deshpande[1,2], Manoj Gopalkrishnan[3], Thomas E. Ouldridge[4], and Nick S. Jones[1]

[1] *Department of Mathematics, Imperial College London, London SW7 2AZ, United Kingdom*
[2] *School of Technology and Computer Science, Tata Institute of Fundamental Research, Mumbai 400005, India*
[3] *Department of Electrical Engineering, Indian Institute of Technology Bombay, Mumbai 400076, India*
[4] *Department of Bioengineering, Imperial College London, London SW7 2AZ, United Kingdom*

1 Validating the timestep of the integrator

We validate the accuracy of our Langevin integrator by considering the dependence of thermodynamic expectations on the time step. We calculate the average potential and kinetic energies for a particle in a quadratic potential $W_{A,B} = A\left(\frac{x}{B} - 1\right)^2$, a quadratic proxy for a single well of the quartic resting-state potential. We plot the results in Figure S1 for a few representative values of the friction coefficient $\gamma = [0.1, 1.10, 100]$ and $A = 10$. Each result is based on an average from 10 simulations each of 5×10^8 time steps. As is evident from the figure, a time step of 0.001 gives good convergence to the equipartition limit of $k_BT/2$.

However, it is not sufficient to just compare the average kinetic and potential energies to the equipartition limit. We need to ensure that the observed kinetics are robust to our choice of time step. In particular, we need to test that a time step of 0.001 is sufficient for the highest values of our control parameter F, which presents the most severe challenge to integrating our Langevin equation (due to the behaviour near $x = 0$). Figure S2 confirms that a time step of 0.001 is appropriate for $F = 100$ and the full range of γ tested. Each value in the figure is an average over 1000 initial conditions.

2 Erasure region

In this section, we demonstrate that our results are not limited to our specific definition of the erasure region by considering two alternative criteria for erasing and confirming that our earlier conclusions are supported.

2.1 Accuracy of erasure: Convergence of probability distribution

Recall that the erasing time is a sum of transport time (τ_t) and the mixing time (τ_m). Since the transport time is independent of the metric used to measure the mixing time, we analyse

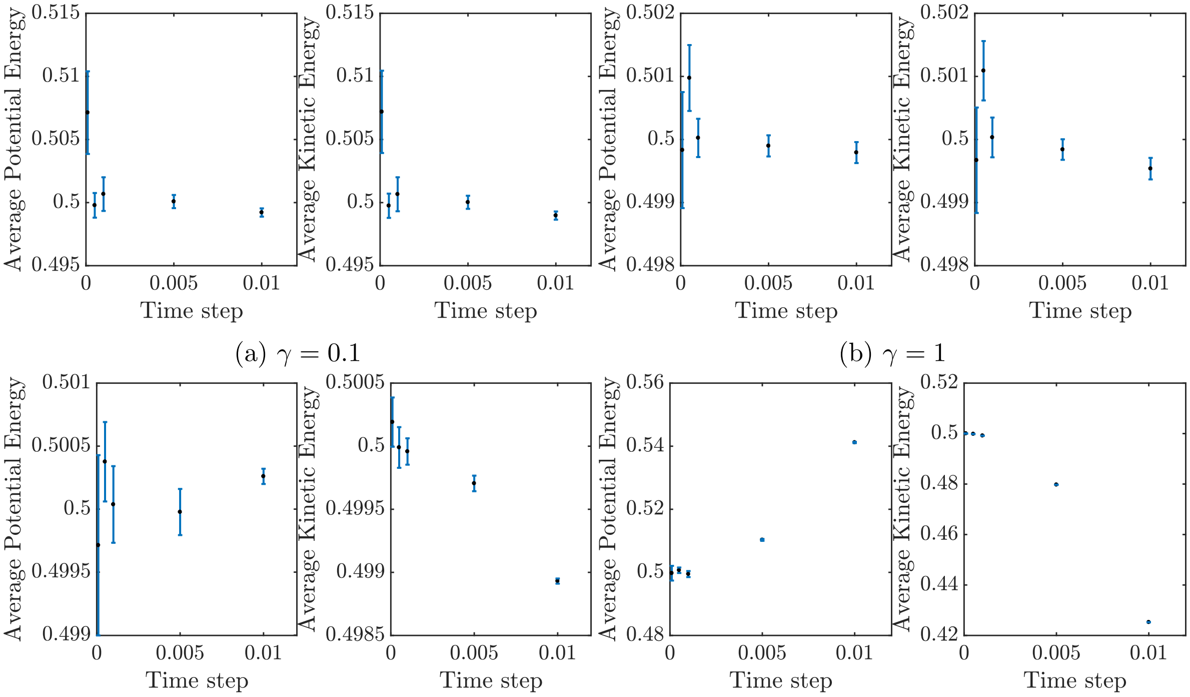

(a) $\gamma = 0.1$ (b) $\gamma = 1$

(c) $\gamma = 10$ (d) $\gamma = 100$

FIGURE S1: A time step of 0.001 is good enough to ensure that the average potential and kinetic energies approaches $\frac{k_B T}{2} = 0.5$ for a wide range of friction values.

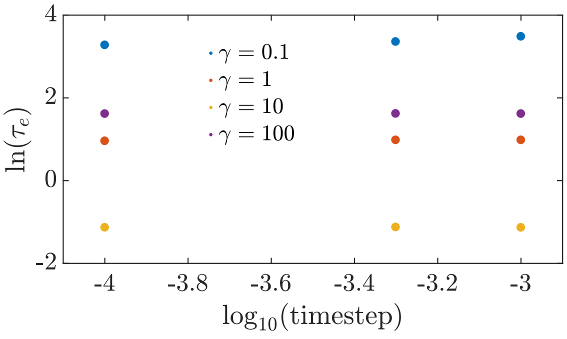

FIGURE S2: A time step of 0.001 gives reasonable values of erasing time for the largest value of the control parameter ($F = 100$) that we use in the simulations.

a proxy for the mixing process in isolation. Specifically, we consider the relaxation of a particle in a harmonic well, initially prepared in an arbitrary non-equilibrium distribution. As an alternative definition of mixing, we consider τ_m^ϵ as the first time when the probability distribution of the particle comes within a certain distance (in the appropriate norm, and relative to its initial distribution) of the Gibbs distribution corresponding to the well. More specifically,

$$\tau_m^\epsilon = \inf_{t\geq 0}\{||\text{law}((x(t),p(t)) - \pi_0(x,p)||_{L^2(\pi_0(x,p))} \leq \epsilon||\text{law}((x(0),p(0)) - \pi_0(x,p)||_{L^2(\pi_0(x,p))}\}$$

where $(x(t),p(t))$ is the solution to Equation 7 given appropriate initial conditions. Here, $\pi_0(x,p)$ is the stationary distribution of the harmonic well. As is usual, the weighted norm $L^2(\pi_0(x,p)) := \{f| \int_{-\infty}^{\infty}\int_{-\infty}^{\infty}|f|^2\pi_0(x,p)dxdp < \infty\}$. Informally, τ_e^ϵ is the time required for the distribution to be a factor $\epsilon \ll 1$ "closer" to the equilibrium distribution than in the initial condition.

We define $\eta = 1-\epsilon$ as the **accuracy** of erasure. Lesser the ϵ, the closer the distribution of the particle is to the Gibbs distribution of the harmonic well and hence more accurate the erasure.

Consider the modified Langevin equation

$$\begin{aligned} m\,dx &= p\,dt \\ dp &= -\gamma p\,dt - \partial_x N_{A,B}(x)\;dt + \sqrt{2m\gamma k_B T}\,dW \end{aligned} \tag{1}$$

Here $N_{A,B}(x) = \frac{1}{2}m\omega_0^2(x-B)^2$ where $\omega_0 = \sqrt{\frac{8A}{mB^2}}$ is the harmonic potential that approximates well "0". Equation1 has the generator [2, pp. 182] given by

$$\mathcal{L} = \frac{p}{m}\partial_x - (\partial_x N_{A,B}(x))\,\partial_p + \gamma\left(-p\partial_p + k_B T\partial_p^2\right) \tag{2}$$

It is common knowledge that the following equation is true [1].

$$||\text{law}((x(t),p(t)) - \pi_0(x,p)||_{L^2(\pi_0(x,p))} \leq e^{-\lambda t}||\text{law}((x(0),p(0)) - \pi_0(x,p)||_{L^2(\pi_0(x,p))}$$

where λ is the first non-zero eigenvalue of the generator $\mathcal{L}$ given by Equation 2. Setting $e^{-\lambda t} = \epsilon$, we get useful **upper bounds** on the mixing time. In particular, we get

$$\tau_m^\epsilon \leq \frac{1}{\lambda}\ln\frac{1}{\epsilon} \tag{3}$$

For the sake of rough scaling, we will use $\tau_m^\epsilon \approx \frac{1}{\lambda}\ln\frac{1}{\epsilon}$ as an approximate estimate of the mixing time. It is important to note that the generator $\mathcal{L}$ is not self-adjoint and may possess imaginary eigenvalues. The rate of convergence in such cases will be determined by the real part of the eigenvalue. In fact using [2, pp. 200], the first non-zero eigenvalue of the generator is

$$\lambda = \frac{\gamma}{2} - \frac{1}{2}\sqrt{\gamma^2 - 4\omega_0^2}$$

In the underdamped limit when $\gamma \ll 2\omega_0$, we have $Re(\lambda) = \frac{\gamma}{2}$. Therefore $\tau_m^\epsilon \approx \frac{2}{\gamma}\log(\frac{1}{\epsilon})$ in the low friction regime. When friction is very high i.e. $\gamma \gg \omega_0$, we have $\lambda \approx \frac{\omega_0^2}{\gamma}$. As

a result we get $\tau_m^\epsilon \approx \frac{\gamma}{\omega_0^2}\log\frac{1}{\epsilon}$. Thus our proxy for the mixing process produces $\tau_m^\epsilon \propto \frac{1}{\gamma}$ in the low friction regime and $\tau_m^\epsilon \propto \gamma$ in the high friction regime, consonant with the scaling and non-monotonicity observed using the erasure region criterion. As a consequence, using a convergence criterion for erasure would not change the physics of the problem, merely perturbing the erasing time-scale quantitatively.

2.2 $4k_BT$ criterion for erasure region

Within the framework of the original "erasure region" criterion discussed in the main text, we now consider the robustness of results to changing the numerical value of the criterion. Specifically, we here define the erasure region as all phase space points with total energy atleast $4k_BT$ below the barrier height. More formally,

$$\tau_e^{4k_BT} = \mathbb{E}\left[\inf\{t \geq 0 \mid x(t) < 0 \text{ and } H(x(t), p(t)) \leq A - 4k_BT\}\right]$$

where $(x(t), p(t))$ is the solution to Equation 7 with the initial condition $(x(0), p(0)) \sim_{\text{law}} \pi_1(x,p)$. We now show that we get the same non-monotonicity and scaling of erasing time as a function of friction-coefficient that we got using the $3k_BT$ criterion. In particular, the erasing time scales as $\frac{1}{\gamma}$ in the low friction regime and scales as γ at high friction. Figure S3 illustrates this fact. Fits are performed using analytical expressions equivalent to those discussed in the main text, but adjusted for the new numerical value of the boundary of the erasure region.

1. Low friction regime:

$$\tau_e^{4k_BT} \approx \sqrt{\frac{2mB}{F}} + \frac{1}{\gamma}\ln\frac{A + F\cdot B}{A - 4k_BT}. \tag{4}$$

2. High friction regime:

$$\tau_e^{4k_BT} \approx \frac{mB\gamma}{F} + \frac{2mB^2\gamma}{5A} \tag{5}$$

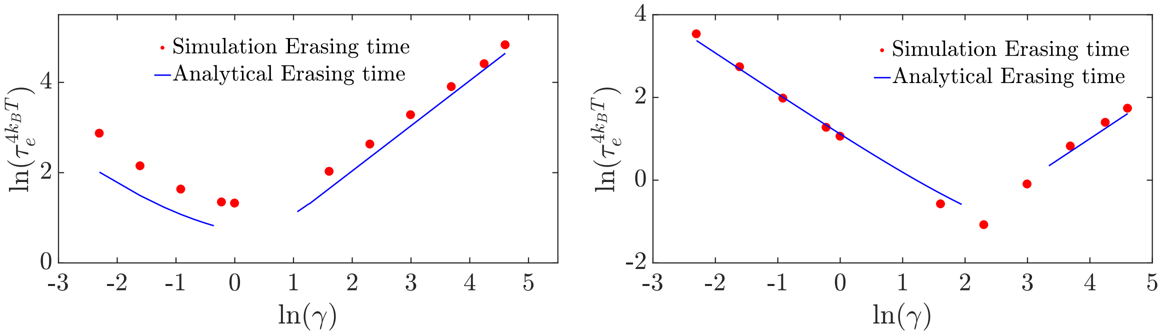

(a) $A = 10, F = 1$

(b) $A = 10, F = 100$

FIGURE S3: Evidence from simulation that the use of $4k_BT$ to define the erasure reason does not change the fundamental physics of the problem.

3 Work calculation

3.1 Full calculation for work done at t=0

We first provide a more detailed justification of the approximation $W = (A + F \cdot B - k_B T/2)/2$ for the work done when the control potential is switched on, then compare it to the exact result. From Equation 12 , and letting $I = \{x \geq 0 \mid A - U_{A,B}(x) + F \cdot x \geq 0\}$, we get

$$\begin{aligned}\langle W \rangle &= \int_0^\tau \int_I V_F(x,t)\, p(x,t)\, \delta(t)\, dx dt \\ &= \int_I (A - U_{A,B}(x) + F \cdot x)\, p(x,0)\, dx \end{aligned} \tag{6}$$

Since $p(x,0) \propto \mathrm{e}^{-\frac{U_{A,B}(x)}{k_B T}}$, we can rewrite the expression of work as

$$\langle W \rangle = \frac{\int_I (A - U_{A,B}(x) + F \cdot x)\, \mathrm{e}^{-\frac{U_{A,B}(x)}{k_B T}}\, dx}{\int_{-\infty}^{\infty} \mathrm{e}^{-\frac{U_{A,B}(x)}{k_B T}}\, dx} \tag{7}$$

Since $I \subseteq [0, \infty)$ and $(A - U_{A,B}(x) + F \cdot x)\, \mathrm{e}^{-\frac{U_{A,B}(x)}{k_B T}}$ is negligible as $x \to \infty$, replacing the upper limit of integration by ∞ is reasonable. Hence the integral becomes

$$\langle W \rangle \approx \frac{\int_0^{\infty} (A - U_{A,B}(x) + F \cdot x)\, \mathrm{e}^{-\frac{U_{A,B}(x)}{k_B T}}\, dx}{\int_{-\infty}^{\infty} \mathrm{e}^{-\frac{U_{A,B}(x)}{k_B T}}\, dx} \tag{8}$$

When $A >> k_B T$, we can use Bessel's functions to approximate this integral giving

$$\langle W \rangle \approx \frac{\left(A + F \cdot B - \frac{k_B T}{2}\right)}{2} \tag{9}$$

justifying the crude approximation in the main text. The accuracy of this expression compared to Eq. 7 is illustrated in Figure S4.

3.2 The potential for energy recovery is negligible

Here, we argue that energy recoverable at the end of the protocol is very small, and hence may be neglected. We assume that the control is switched off after a time τ sufficiently large compared to τ_e so that the proportion of particles remaining on the right hand side of the well is determined by the Boltzmann factor. The work that we could then in principle recover is given by the following expression:

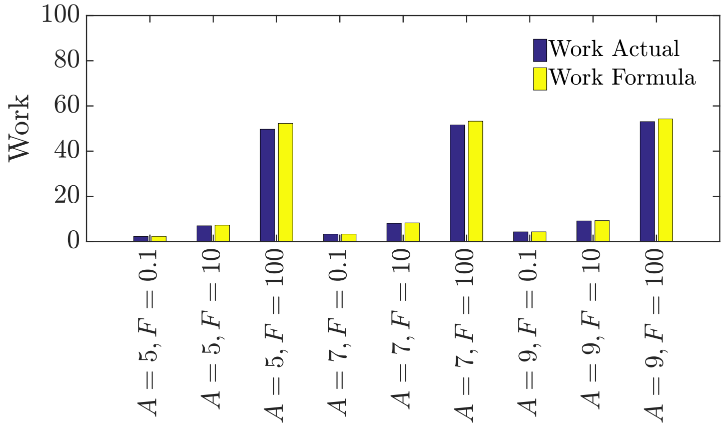

FIGURE S4: Work when the control is switched on against various values of A and F, comparing the full expression Eq. 7 and the approximate result Eq. 9.

$$\langle W_{rec} \rangle \approx \frac{\int_I \left(A - U_{A,B}(x) + F\cdot x\right) e^{\frac{-(A+F\cdot x)}{k_B T}}\, dx}{\int_{-\infty}^{0} e^{\frac{-U_{A,B}(x)}{k_B T}}\, dx + \int_0^{\infty} e^{\frac{-(A+F\cdot x)}{k_B T}}\, dx} \tag{10}$$

Recall that $I = \{x \geq 0 \mid A - U_{A,B}(x) + F\cdot x \geq 0\}$. This implies that $I = [0, x^*]$, where $A - U_{A,B}(x^*) + F\cdot x^* = 0$. Using Equations 9 and 10, we will calculate the fraction of recovered work i.e., $W^f_{rec} = \frac{\langle W_{rec}\rangle}{\langle W\rangle}$. Figure S5 precisely calculates this quantity. As is evident from the figure, the fraction is almost negligible and reaches its maximum value at low A and F.

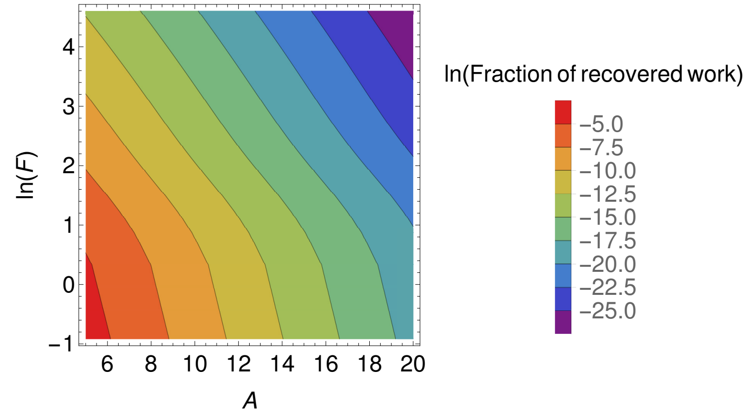

FIGURE S5: Negligible energy can be recovered for our family of controls.

4 Regression and Cross Validation

We use cubic regression to interpolate between simulation data for both the reliability and erasing time-scales. Let $F' = \log(F)$ and $\gamma' = \log(\gamma)$. Then we use the following polynomials to fit the time-scales.

1. **Erasing Polynomial**:

$$\begin{aligned}\log(\tau_e) = b_1 + b_2A^3 + b_3F'^3 + b_4\gamma'^3 + b_5A^2F' + b_6A'F'^2 + b_7F'^2\gamma' + b_8F'\gamma'^2 \\ + b_9A'^2\gamma' + b_{10}A\gamma'^2 + b_{11}A^2 + b_{12}F'^2 + b_{13}\gamma'^2 + b_{14}AF' + b_{15}F'\gamma' \\ + b_{16}A\gamma' + b_{17}A + b_{18}F' + b_{19}\gamma'\end{aligned} \tag{11}$$

2. **Reliability Polynomial**:

$$\log(\tau_r) = c_1 + c_2A^3 + c_3\gamma'^3 + c_4A^2\gamma' + c_5A\gamma'^2 + c_6A^2 + c_7\gamma'^2 + c_8A\gamma' + c_9A + c_{10}\gamma \tag{12}$$

, where the coefficients $b_1, b_2, \cdots b_{19}$ and $c_1, c_2, \cdots c_{10}$ are to be determined by regression.

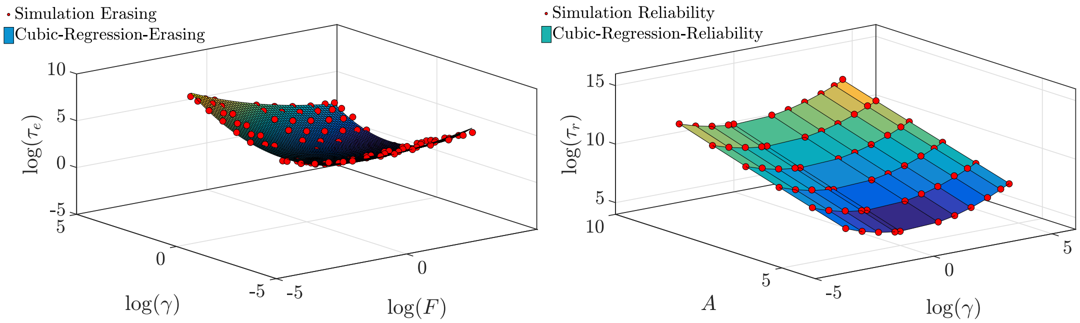

(a) Erasing time from simulation and cubic-regression for $A = 6$

(b) Reliability time from simulation and cubic-regression

FIGURE S6

Figure S6 gives a visual illustration of the fact that cubic fits offer a good approximation to the simulation results for both the erasing and reliability time-scales. In what follows, we present a more detailed and formal justification using cross-validation.

We perform “Leave-one-out” cross validation to justify the use of cubic regression. Figure S7 reports the mean square training and testing cross-validation errors corresponding to linear, quadratic and cubic fits. A lower value of the testing error indicates a good fit. Cubic regression has the lowest value of testing errors amongst the fits considered for both the reliability and erasing time-scales. Figure S7 confirms that the training and testing errors corresponding to cubic-regression for both the time-scales are roughly comparable(with the training error being slightly lower than the testing error). As a result, we can safely assume that the cubic polynomial does not over-fit the data and use it for modelling both the time-scales.

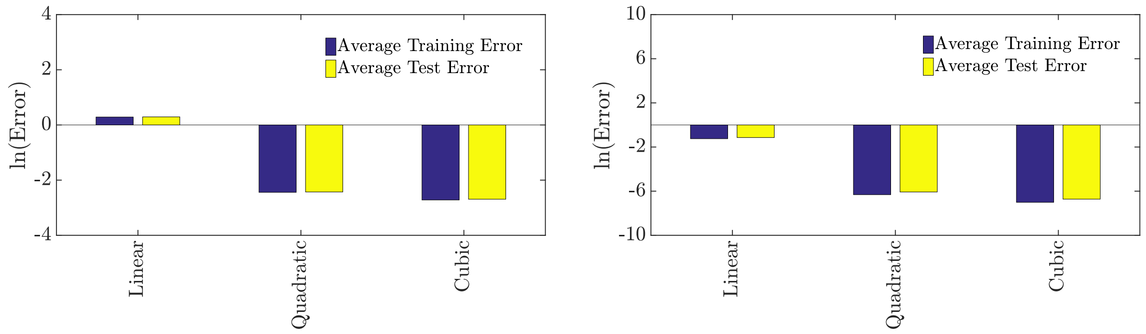

(a) Errors of regression fits for erasing time

(b) Errors of regression fits for reliability time

FIGURE S7

5 Calculation of well parameters

Here we calculate the quantities needed to apply Equation 13 to our system, with the potential $U_{A,B}(x) = A\left(\frac{x^2}{B^2}-1\right)^2$. We have $\partial_x U_{A,B}(x) = \frac{4Ax\left(x^2-B^2\right)}{B^4}$ and $\partial_{xx} U_{A,B}(x) = \frac{4A\left(3x^2-B^2\right)}{B^4}$.

1. **Angular frequency at barrier height** (ω_b): We can approximate the region near the barrier by an inverted harmonic oscillator. By Taylor expanding the potential about the point $x=0$, we get

$$U_{A,B}(x) \approx U(0) + \partial_x U_{A,B}(x)\Big|_{x=0} x + \frac{\partial_{xx} U_{A,B}(x)\Big|_{x=0} x^2}{2} \approx A - \frac{2Ax^2}{B^2} = A - \frac{m\omega_b^2 x^2}{2} \quad (13)$$

Therefore we have $\omega_b = \sqrt{\frac{4A}{mB^2}}$.

2. **Angular frequency at the bottom of the well** (ω_0): We can approximate the region near the bottom of the well by a harmonic oscillator. By Taylor expanding the potential about the point $x=B$, we get

$$\begin{aligned} U_{A,B}(x) &\approx U(B) + \partial_x U_{A,B}(x)\Big|_{x=B}(x-B) + \frac{\partial_{xx} U_{A,B}(x)\Big|_{x=B}(x-B)^2}{2} \\ &= \frac{4A(x-B)^2}{B^2} = \frac{m\omega_0^2 (x-B)^2}{2} \end{aligned} \quad (14)$$

Therefore we have $\omega_0 = \sqrt{\frac{8A}{mB^2}}$.

3. **Action at barrier height** $I(A)$: Consider a particle of mass m with a starting velocity $v=0$, moving along a constant energy surface with energy A. The particle starts at $x=0$ and moves to $x=\sqrt{2}B$ and returns back to $x=0$. The action for this round trip is given by $I(A) = \oint p\,dx = 2\sqrt{2m}\int_0^{\sqrt{2}B}\sqrt{A - A\left(\frac{x^2}{B^2}-1\right)^2}\,dx = \frac{8B\sqrt{mA}}{3}$.

6 Locally trapped bits are uniquely trapped

In this section, we give typical plots for our family of controls that show no evidence of multiple local minima in erasing time within a level set of work. Towards this we let $F' = \log(F)$ and $\gamma' = \log(\gamma)$. Using the same form of regression polynomial as in Equation 11, but at constant work W, this translates to

$$\begin{aligned}\log(\tau_e) = b_1 &+ b_2(W - e^{F'})^3 + b_3F'^3 + b_4\gamma'^3 + b_5(W - e^{F'})^2F' + b_6(W - e^{F'})F'^2 + b_7F'^2\gamma' + b_8F'\gamma'^2 \\ &+ b_9(W - e^{F'})^2\gamma' + b_{10}(W - e^{F'})\gamma'^2 + b_{11}(W - e^{F'})^2 + b_{12}F'^2 + b_{13}\gamma'^2 + b_{14}(W - e^{F'})F' \\ &+ b_{15}F'\gamma' + b_{16}(W - e^{F'})\gamma' + b_{17}(W - e^{F'}) + b_{18}F' + b_{19}\gamma'\end{aligned} \tag{15}$$

Note that $\left(\frac{d\tau_e}{d\gamma'}\right)_{W,F'} = \gamma\left(\frac{d\tau_e}{d\gamma}\right)_{W,F}$ and $\left(\frac{d\tau_e}{dF'}\right)_{W,\gamma'} = F\left(\frac{d\tau_e}{dF}\right)_{W,\gamma}$. Therefore solving for $\left(\frac{d\tau_e}{d\gamma}\right)_{W,F} = \left(\frac{d\tau_e}{dF}\right)_{W,\gamma} = 0$ is equivalent to solving for $\left(\frac{d\tau_e}{d\gamma'}\right)_{W,F'} = \left(\frac{d\tau_e}{dF'}\right)_{W,\gamma'} = 0$. We solve $\left(\frac{d\tau_e}{d\gamma'}\right)_{W,F'} = \left(\frac{d\tau_e}{dF'}\right)_{W,\gamma'} = 0$ numerically and plot it in Figure S8. As illustrated by Figure S8, there is exactly one solution to the equations $\left(\frac{d\tau_e}{d\gamma}\right)_{W,F} = \left(\frac{d\tau_e}{dF}\right)_{W,\gamma} = 0$ within the broad range of parameters allowed, confirming our assumption that locally trapped bits are uniquely trapped.

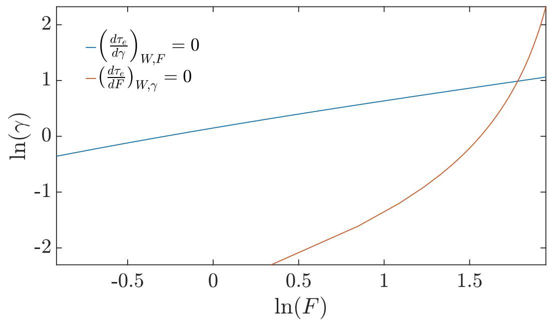

(a) W=12

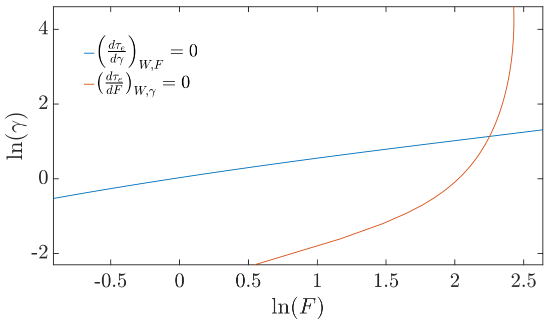

(b) W=19

FIGURE S8: Evidence that for our family of controls, locally trapped bits are uniquely trapped. The system $\left(\frac{d\tau_e}{d\gamma}\right)_{W,F} = \left(\frac{d\tau_e}{dF}\right)_{W,\gamma} = 0$ has exactly one solution within the broad range of parameters considered, which corresponds to a unique local minimum of erasing time in a level set of work. This is illustrated for work $W = 12$ and $W = 19$. The situation is representative for other values of work.

7 Geometry of the Optimal Bit

1. **Proof of Claim 4.1**

Proof. (a) For contradiction suppose that $\tau_e(A, F, \gamma) < t_e$. Since τ_e is continuous and it is possible to locally decrease work at fixed reliability time, there exists a design

(A', F', γ') with $W(A', F', \gamma') < W(A, F, \gamma)$ that is (t_r, t_e)-feasible contradicting the optimality of the design (A, F, γ).

(b) For contradiction suppose that $\tau_r(A, F, \gamma) > t_r$. Since the design (A, F, γ) is not locally trapped and τ_r is continuous, there exists a design (A_0, F_0, γ_0) requiring work $W(A_0, F_0, \gamma_0) = W(A, F, \gamma)$, erasing time $\tau_e(A_0, F_0, \gamma_0) < \tau_e(A, F, \gamma) \leq t_e$ and maintaining reliability time $\tau_r(A_0, F_0, \gamma_0) \geq t_r$. Thus the design (A_0, F_0, γ_0) is (t_r, t_e)-optimal contradicting Claim 4.1.1 that the optimal design saturates the bound on the erasing time constraint.

□

2.

Observation 7.1. The erasing time of trapped designs is a strictly decreasing function of work for our family of protocols (Example 2.1). In other words, if (A_0, F_0, γ_0) and (A^*, F^*, γ^*) are trapped designs with $W(A^*, F^*, \gamma^*) > W(A_0, F_0, \gamma_0)$ then $\tau_e(A^*, F^*, \gamma^*) < \tau_e(A_0, F_0, \gamma_0)$.

Proof. Since W is a continuous increasing function of A (Observation 2.2), one can choose $A' > A^*$ such that $W(A', F_0, \gamma_0) = W(A^*, F^*, \gamma^*)$. Noting that increasing well height at fixed F and γ decreases erasing time (Observation 3.1), we get $\tau_e(A', F_0, \gamma_0) < \tau_e(A_0, F_0, \gamma_0)$. Using the fact that (A^*, F^*, γ^*) is a trapped design, we get $\tau_e(A^*, F^*, \gamma^*) \leq \tau_e(A', F_0, \gamma_0) < \tau_e(A_0, F_0, \gamma_0)$ establishing the claim. □

3. **Proof of Claim 4.2**

Proof. (a) Since $\tau_r(A^*, F^*, \gamma^*) \geq t_r$ and $\tau_e(A^*, F^*, \gamma^*) = t_e$, the design (A^*, F^*, γ^*) is (t_r, t_e)-feasible. Suppose that the design (A^*, F^*, γ^*) is not (t_r, t_e)-optimal. Then there exists a (t_r, t_e)-feasible design (A', F', γ') such that $W(A', F', \gamma') < W(A^*, F^*, \gamma^*)$. Let (A_0, F_0, γ_0) be a trapped design with $W(A_0, F_0, \gamma_0) = W(A', F', \gamma') < W(A^*, F^*, \gamma^*)$. Then $\tau_e(A_0, F_0, \gamma_0) > \tau_e(A^*, F^*, \gamma^*)$ since the erasing time of trapped bits is a strictly decreasing function of work. Using the fact that (A_0, F_0, γ_0) is a trapped design, we get $\tau_e(A', F', \gamma') \geq \tau_e(A_0, F_0, \gamma_0) > \tau_e(A^*, F^*, \gamma^*) = t_e$, a contradiction since (A', F', γ') is a (t_r, t_e)-feasible design.

(b) Immediate from claim 4.2.1 .

(c) For contradiction suppose that the requirement (t_r, t_e) is unsaturated. Then by claim 4.1.1 there exists a (t_r, t_e)-optimal design (A_0, F_0, γ_0) such that $\tau_r(A_0, F_0, \gamma_0) > t_r$ and $\tau_e(A_0, F_0, \gamma_0) = \tau_e(A^*, F^*, \gamma^*) = t_e$. Since locally trapped designs are uniquely trapped, using claim 4.1.2 , we get that the design (A_0, F_0, γ_0) must be uniquely trapped. Noting that uniquely trapped bits are trapped and using the fact that the erasing time of trapped designs is a strictly decreasing function of work, we get $W(A_0, F_0, \gamma_0) = W(A^*, F^*, \gamma^*)$. This implies that $(A_0, F_0, \gamma_0) = (A^*, F^*, \gamma^*)$, a contradiction since $\tau_r(A_0, F_0, \gamma_0) > t_r \geq \tau_r(A^*, F^*, \gamma^*)$.

□

7.1 Alternative proof via KKT conditions

KKT conditions form the foundation of optimization problems [4, 3]. In order to study the KKT conditions, we consider the optimization problem of finding the design with the lowest work that is (t_r, t_e)-feasible.

Problem 7.2.

$$\begin{aligned}(A^*, F^*, \gamma^*) &= \arg \inf_{A,F,\gamma} W(A, F) \\ t_r - \tau_r(A^*, \gamma^*) &\leq 0 \\ \tau_e(A^*, F^*, \gamma^*) - t_e &\leq 0\end{aligned}$$

In order to state the KKT conditions, we will need the notion of a regular point. The following definition will make this precise.

Definition 7.3 (**Regular point**). Let $Sat(x^*)$ denote the set of gradients of the constraints that are saturated at the point x^*. Then x^* is regular iff $Sat(x^*)$ does not form a linearly dependent set.

Theorem 7.4 (**KKT conditions**). *Let (A^*, F^*, γ^*) be a local optimum of 7.2 and a regular point. Then by [4,* (12.1)*, pp. 95], there exists $\lambda_1^*, \lambda_2^* \in \mathbb{R}_{\geq 0}$ such that*

1. $\nabla W(A^*, F^*, \gamma^*) - \lambda_1^* \nabla \tau_r(A^*, \gamma^*) + \lambda_2^* \nabla \tau_e(A^*, F^*, \gamma^*) = 0.$
2. $\lambda_1^*(t_r - \tau_r(A^*, \gamma^*)) = 0$ *and* $\lambda_2^*(\tau_e(A^*, F^*, \gamma^*) - t_e) = 0.$

Given this powerful theorem 7.4, we are now ready to prove the the same result that we obtained earlier but with the machinery of KKT conditions.

Lemma 7.5. Let us assume that it is always possible to locally decrease work at fixed reliability time. Let (A^*, F^*, γ^*) be a local optimum of 7.2. Then either

1. The design (A^*, F^*, γ^*) saturates the bound on both constraints i.e. $\tau_r(A^*, \gamma^*) = t_r$ and $\tau_e(A^*, F^*, \gamma^*) = t_e$ or
2. The design (A^*, F^*, γ^*) saturates the bound on the erasing time constraint i.e. $\tau_e(A^*, F^*, \gamma^*) = t_e$ but does not saturate the bound on the reliability time constraint i.e. $\tau_r(A^*, \gamma^*) > t_r$ and is locally trapped.

Proof. Consider an optimal design (A^*, F^*, γ^*) such that either it does not saturate the bound on the reliability time constraint i.e. $\tau_r(A^*, \gamma^*) > t_r$ or it does not saturate the bound on the erasing time constraint i.e. $\tau_e(A^*, F^*, \gamma^*) < t_e$. Then we have the following cases:

- Case 1: The design (A^*, F^*, γ^*) saturates the bound on the erasing time constraint, but does not saturate the bound on the reliability time constraint i.e. $\tau_r(A^*, \gamma^*) > t_r$ and $\tau_e(A^*, F^*, \gamma^*) = t_e$. This implies that $\lambda_1^* = 0$. Since only one constraint is active, (A^*, F^*, γ^*) is a regular point. Hence, by Theorem 7.4 on KKT conditions, there exists $\lambda_2^* > 0$ such that $\nabla W(A^*, F^*, \gamma^*) + \lambda_2^* \nabla t_e(A^*, F^*, \gamma^*) = 0$. This implies that (A^*, F^*, γ^*) is a stationary point of erasing time in the level set of it's work $W(A^*, F^*, \gamma^*)$. The fact that this stationary point is actually a local minimum follows from claim 4.1. 2 .

- Case 2: The design (A^*, F^*, γ^*) saturates the bound on the reliability time constraint, but does not saturate the bound on the erasing time constraint i.e. $\tau_r(A^*, \gamma^*) = t_r$ and $\tau_e(A^*, F^*, \gamma^*) < t_e$. This implies that $\lambda_2^* = 0$. Since only one constraint is active, (A^*, F^*, γ^*) is a regular point. Hence, by Theorem 7.4 on KKT conditions, there exists $\lambda_1^* > 0$ such that $\nabla W(A^*, F^*, \gamma^*) = \lambda_1^* \nabla t_r(A^*, \gamma^*)$, a contradiction since $\frac{\partial W}{\partial F} \neq 0$ but $\frac{\partial \tau_r}{\partial F} = 0$.
- Case 3: The design (A^*, F^*, γ^*) does not saturate the bound on either constraints i.e. $\tau_r(A^*, \gamma^*) > t_r$ and $\tau_e(A^*, F^*, \gamma^*) < t_e$. Since no constraint is active we have $\nabla W(A^*, F^*, \gamma^*) = 0$, which is not possible.

□

References

[1] J. Mattingly and A. Stuart, *Geometric ergodicity of some hypo-elliptic diffusions for particle motions*, Markov Processes and Related Fields **8** (2002), no. 2, 199–214.

[2] G. Pavliotis, *Stochastic processes and applications*, Springer, 2014.

[3] T. Rapcsák, *Smooth nonlinear optimization in R^n*, Springer **19** (1997), 376.

[4] S. Wright and J. Nocedal, *Numerical optimization*, Springer Science **35** (1999), 67–68.
