# Supplementary figures and images for: Designing the optimal bit: balancing energetic cost, speed and reliability
Source: Proc Math Phys Eng Sci. 2017 Aug 23;473(2204):20170117. doi: 10.1098/rspa.2017.0117 (PMC5582178; doi:10.1098/rspa.2017.0117)

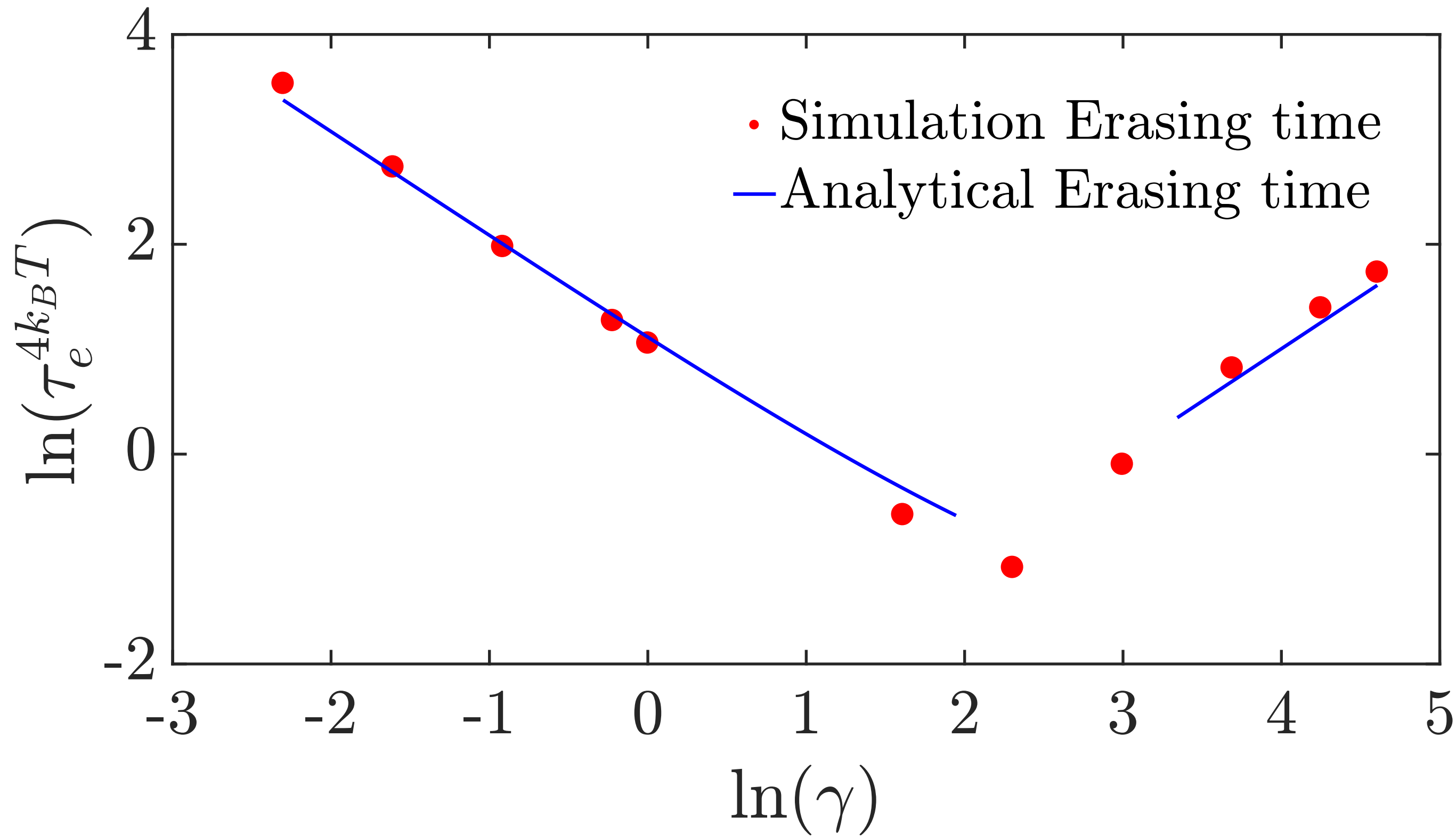

Supplement: Supplementary Information [file rspa20170117supp1.zip › Supplementary/Figures/erasing_F=100_4kT-eps-converted-to.pdf]

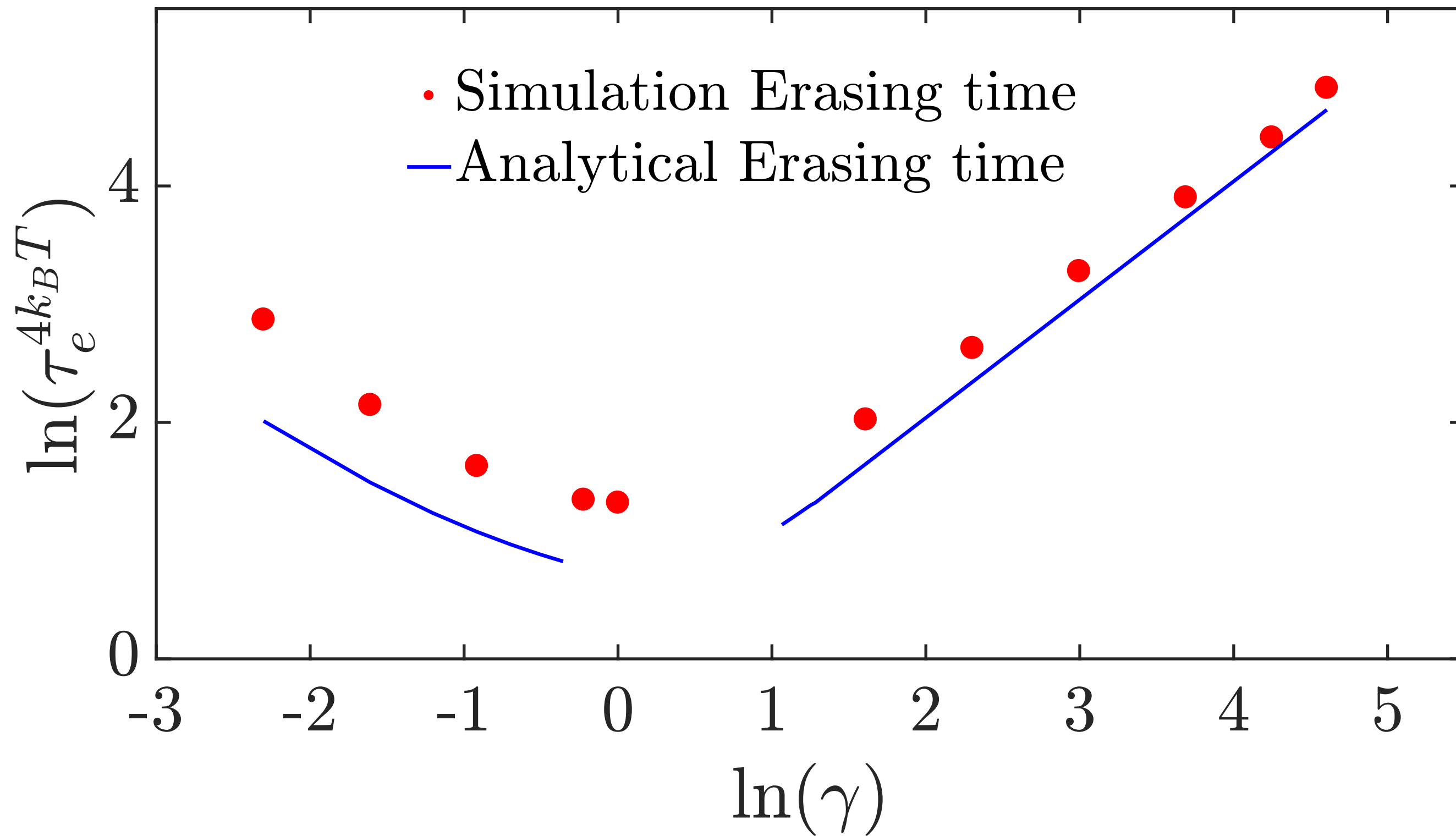

Supplement: Supplementary Information [file rspa20170117supp1.zip › Supplementary/Figures/erasing_F=1_4kT-eps-converted-to.pdf]

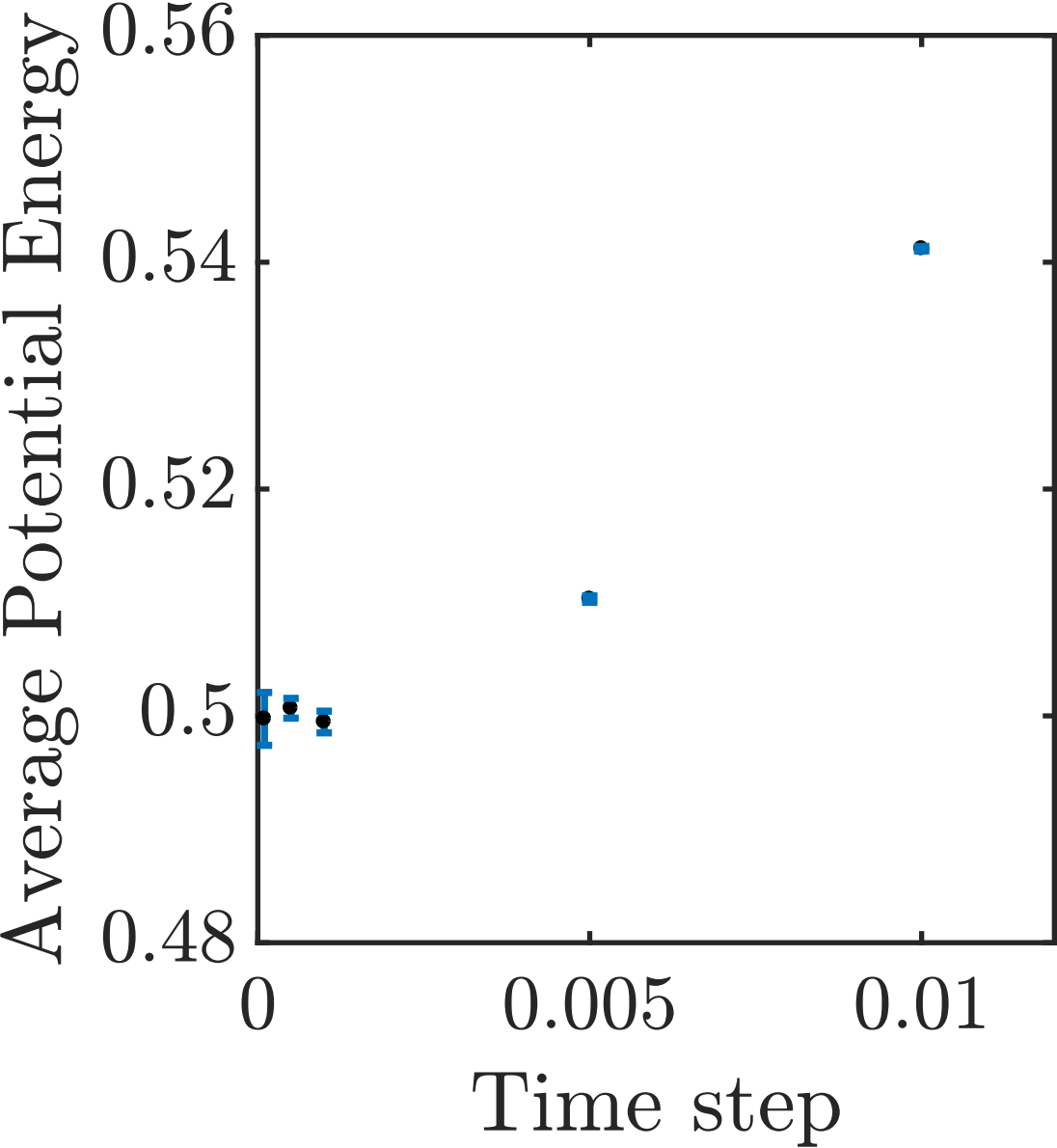

Supplement: Supplementary Information [file rspa20170117supp1.zip › Supplementary/Figures/average_potential_energy_vs_timestep_friction=100-eps-converted-to.pdf]

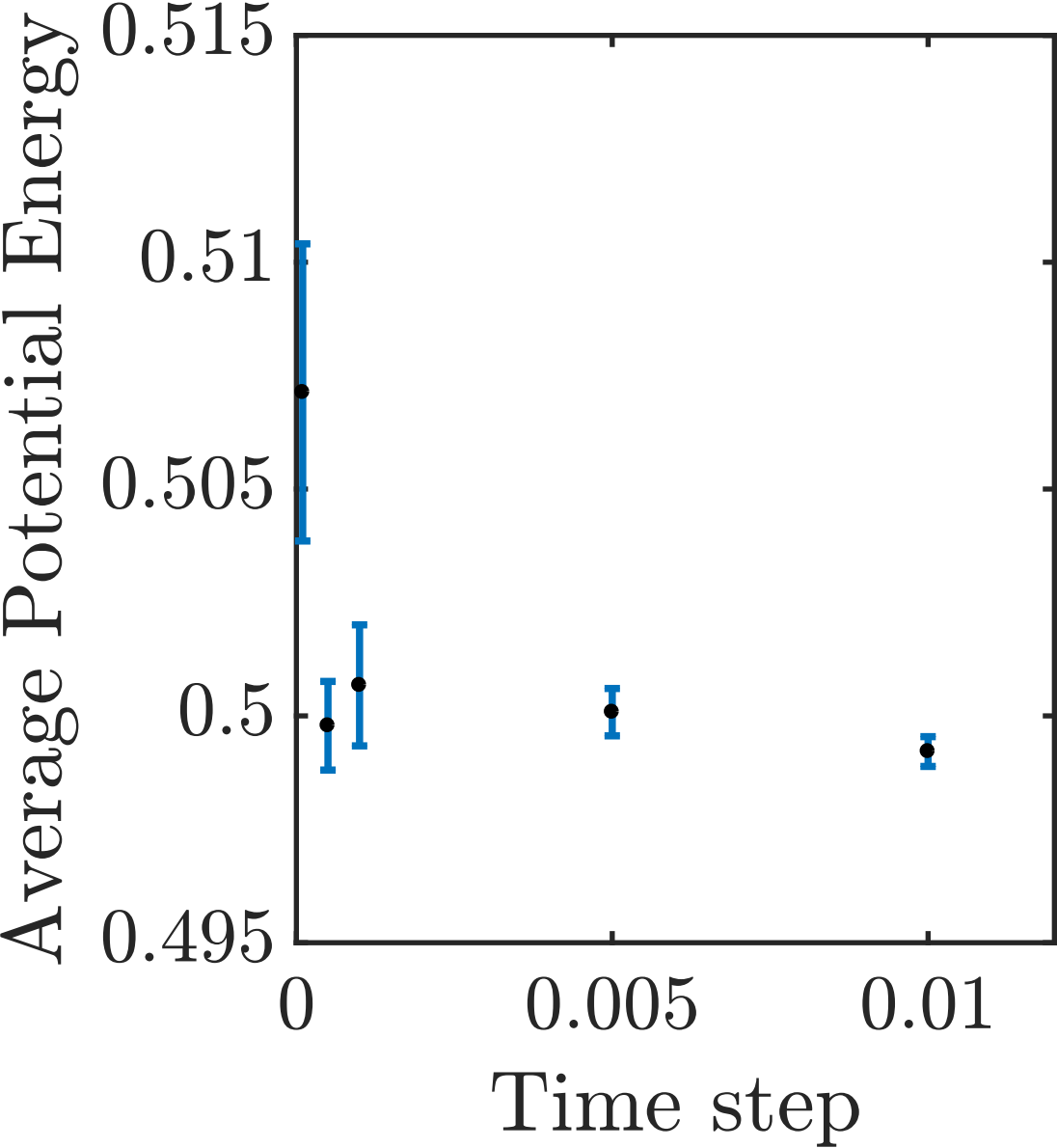

Supplement: Supplementary Information [file rspa20170117supp1.zip › Supplementary/Figures/average_potential_energy_vs_timestep_friction=0_1-eps-converted-to.pdf]

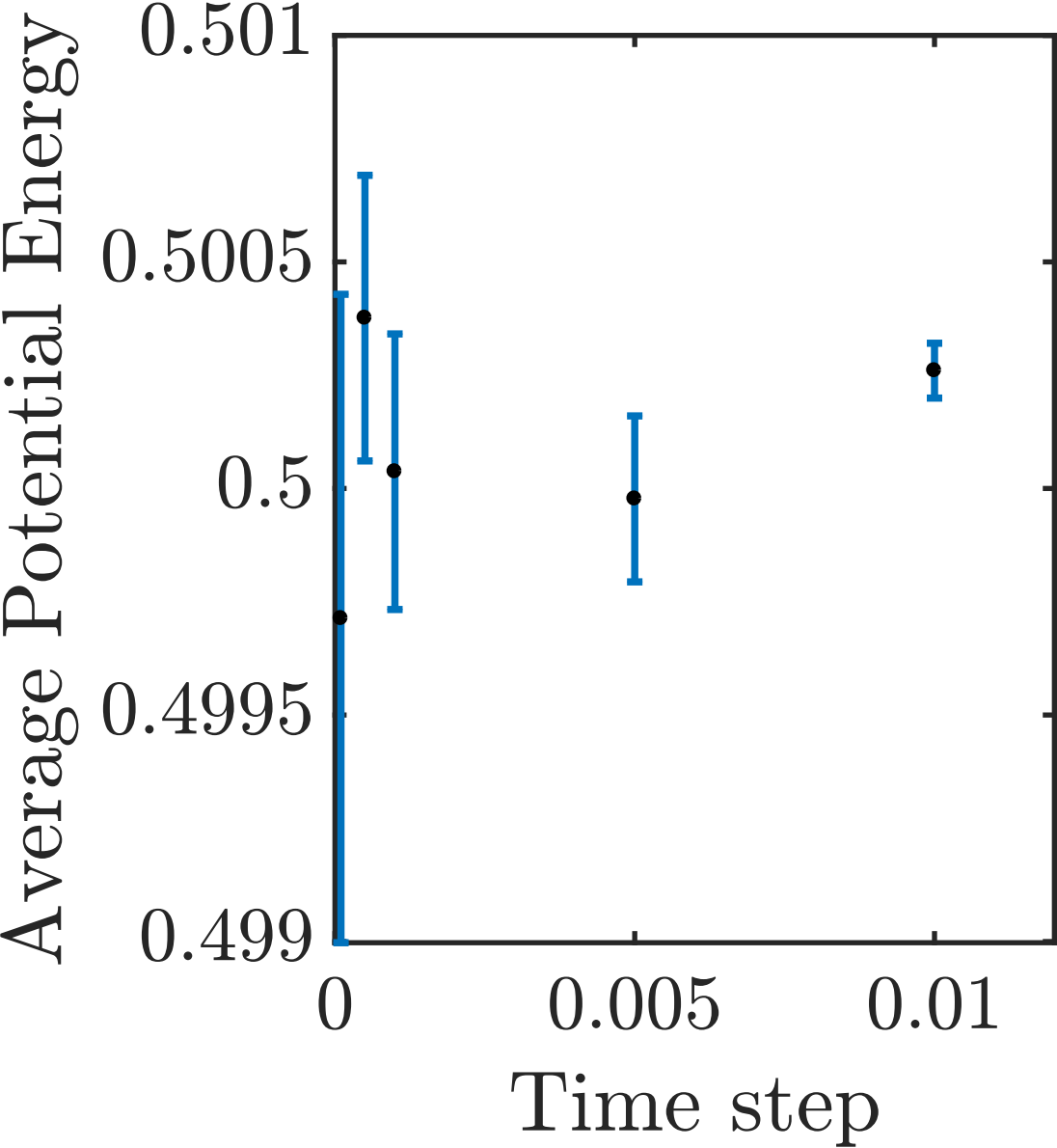

Supplement: Supplementary Information [file rspa20170117supp1.zip › Supplementary/Figures/average_potential_energy_vs_timestep_friction=10-eps-converted-to.pdf]

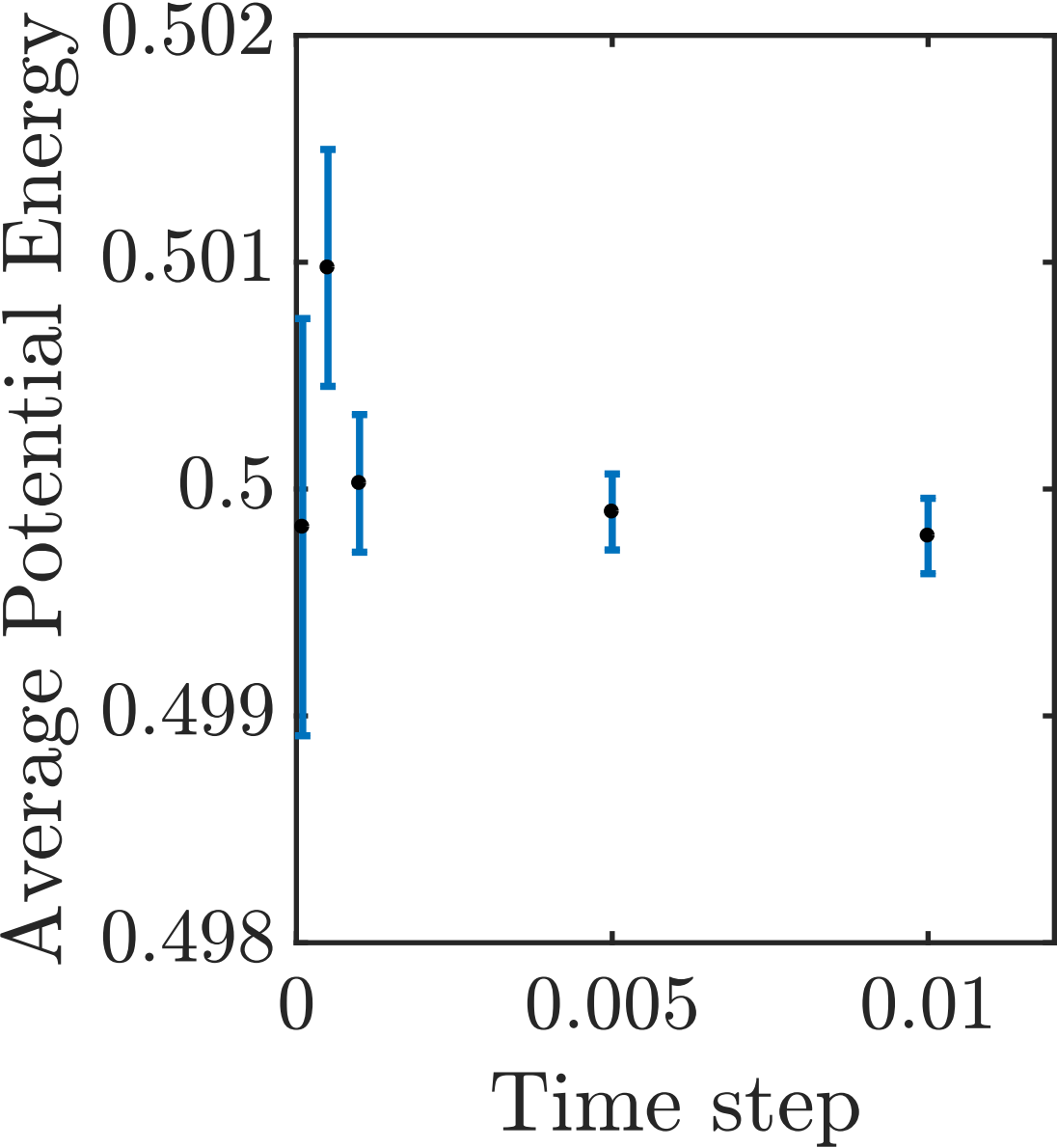

Supplement: Supplementary Information [file rspa20170117supp1.zip › Supplementary/Figures/average_potential_energy_vs_timestep_friction=1-eps-converted-to.pdf]

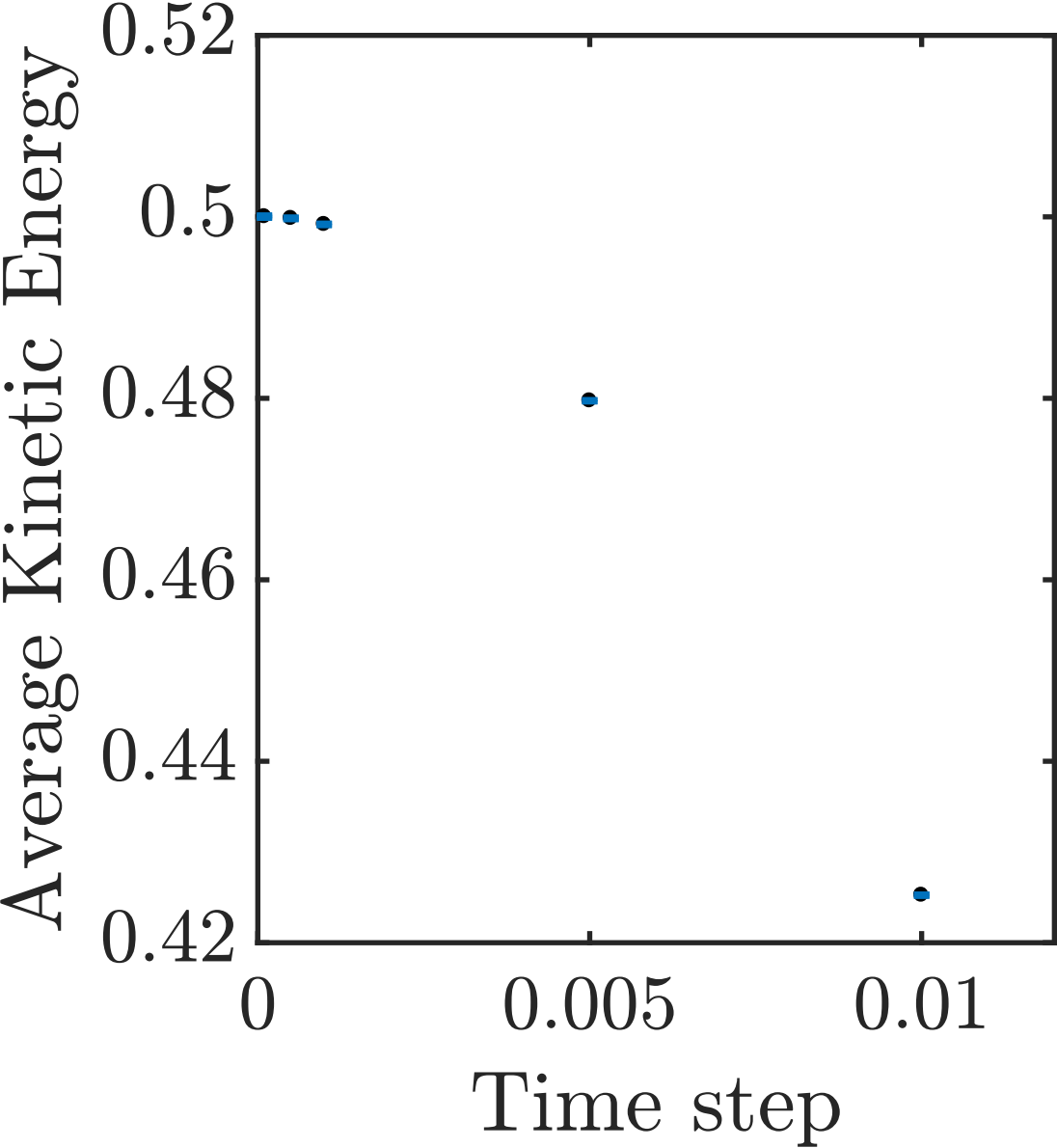

Supplement: Supplementary Information [file rspa20170117supp1.zip › Supplementary/Figures/average_kinetic_energy_vs_timestep_friction=100-eps-converted-to.pdf]

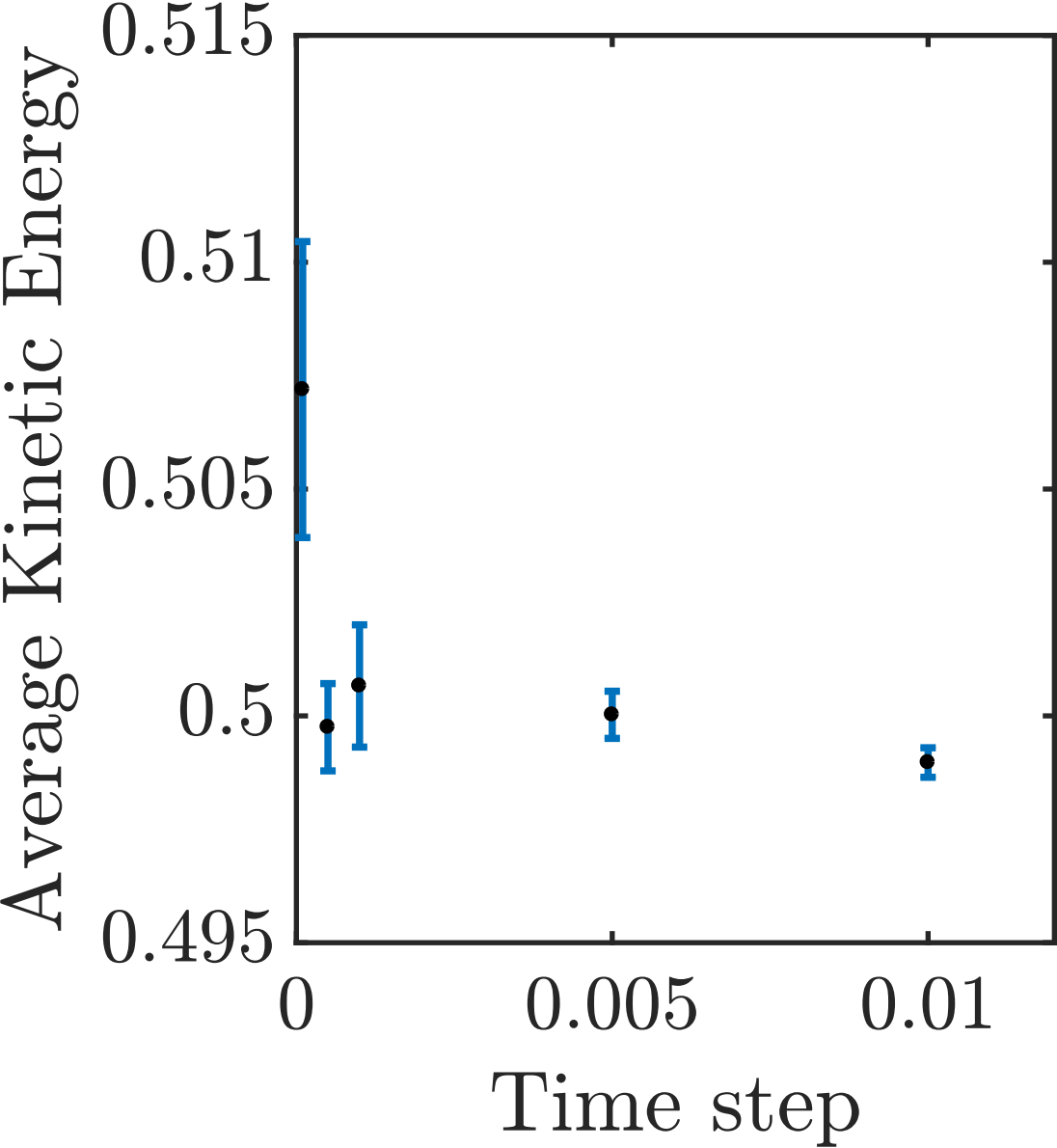

Supplement: Supplementary Information [file rspa20170117supp1.zip › Supplementary/Figures/average_kinetic_energy_vs_timestep_friction=0_1-eps-converted-to.pdf]

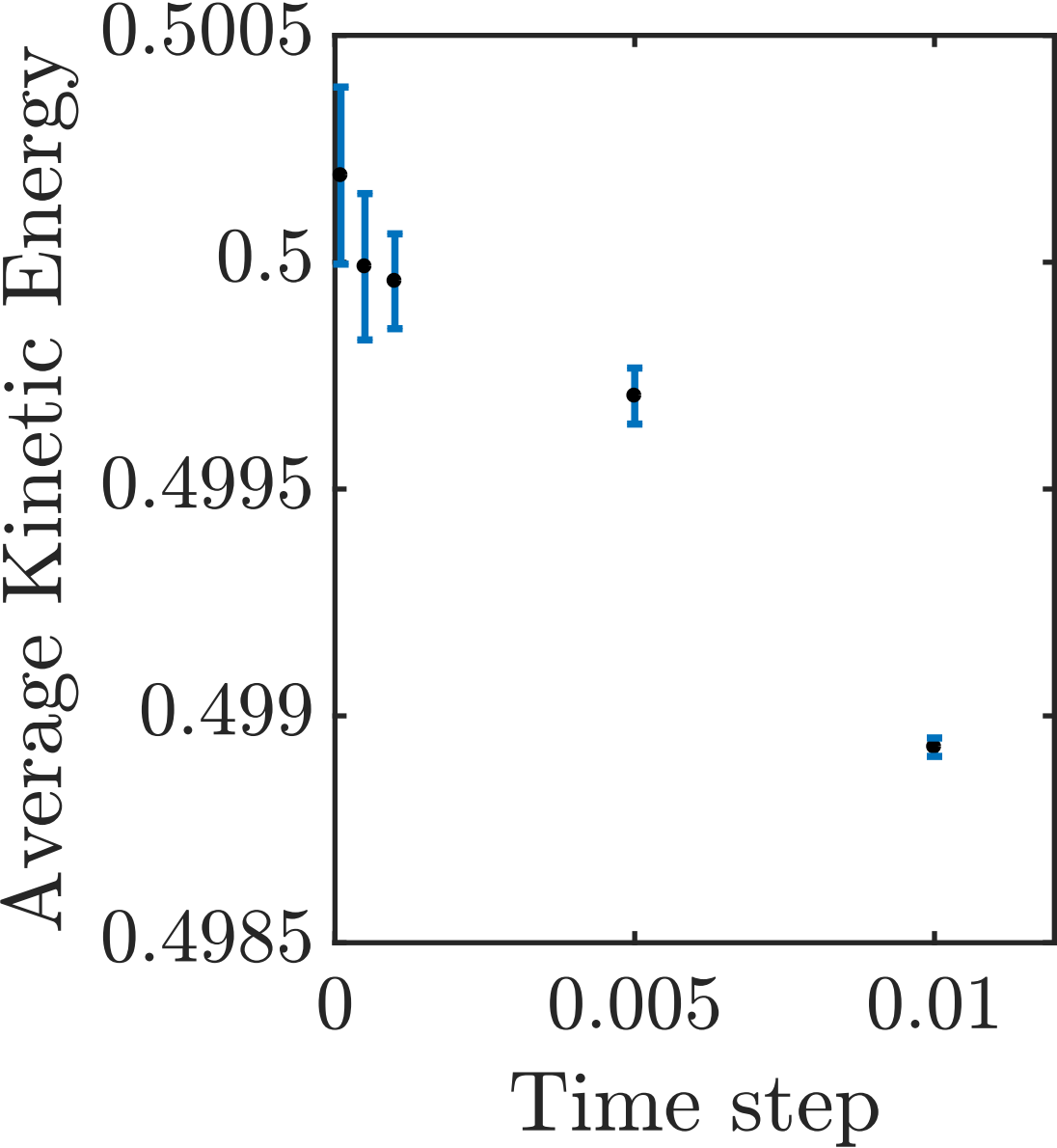

Supplement: Supplementary Information [file rspa20170117supp1.zip › Supplementary/Figures/average_kinetic_energy_vs_timestep_friction=10-eps-converted-to.pdf]

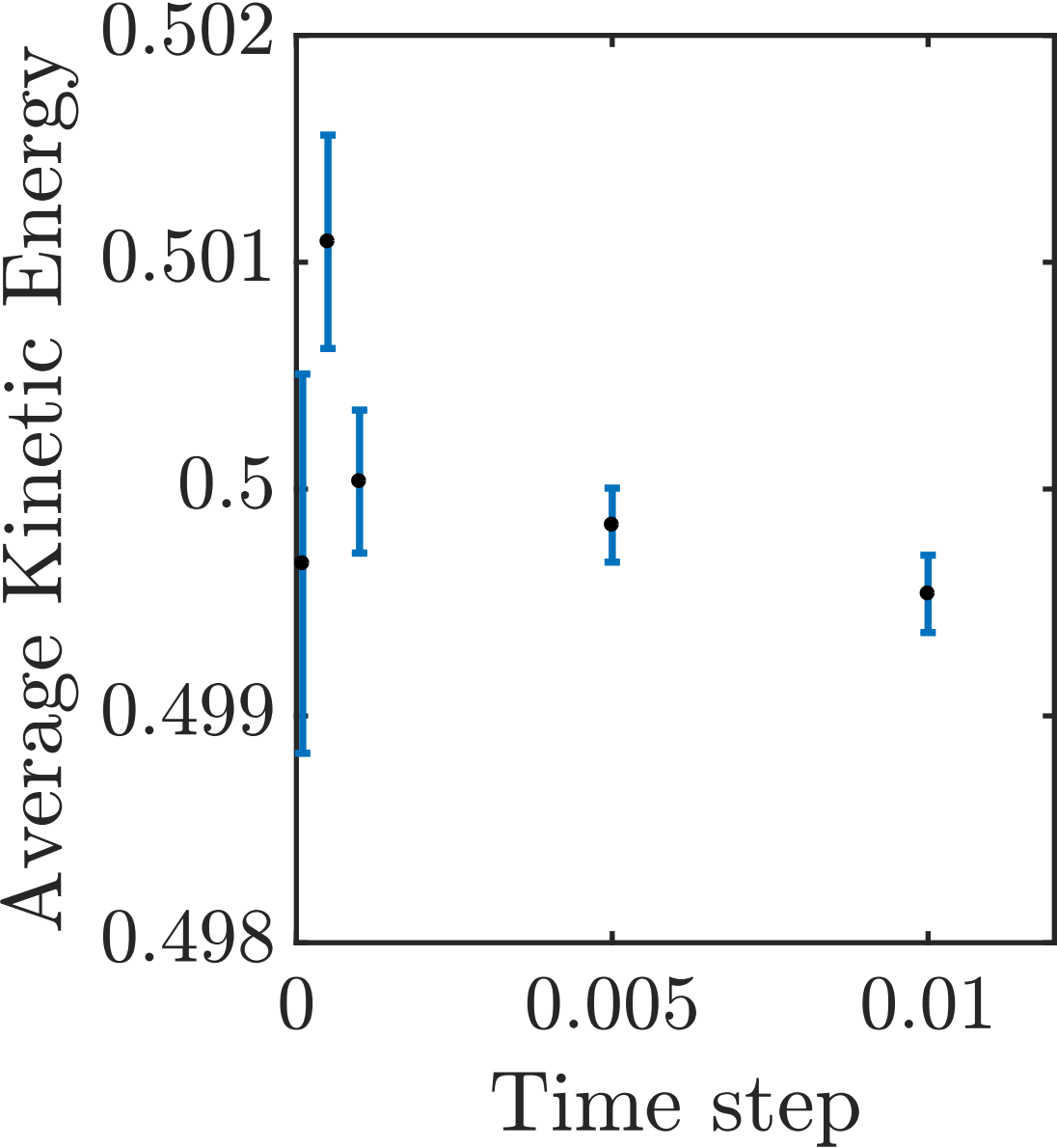

Supplement: Supplementary Information [file rspa20170117supp1.zip › Supplementary/Figures/average_kinetic_energy_vs_timestep_friction=1-eps-converted-to.pdf]

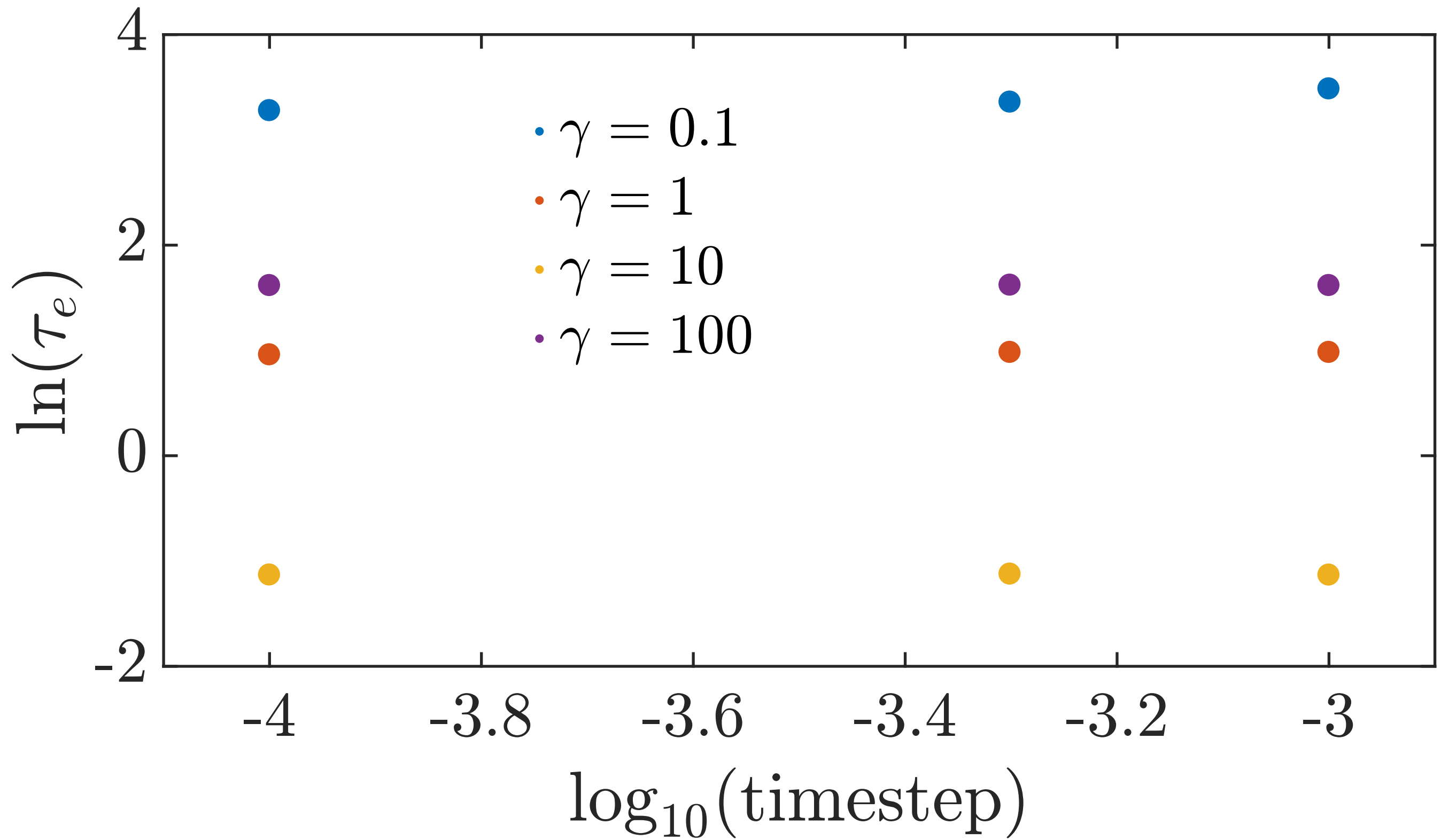

Supplement: Supplementary Information [file rspa20170117supp1.zip › Supplementary/Figures/erasing_time_vs_timestep_A=10_F=100-eps-converted-to.pdf]

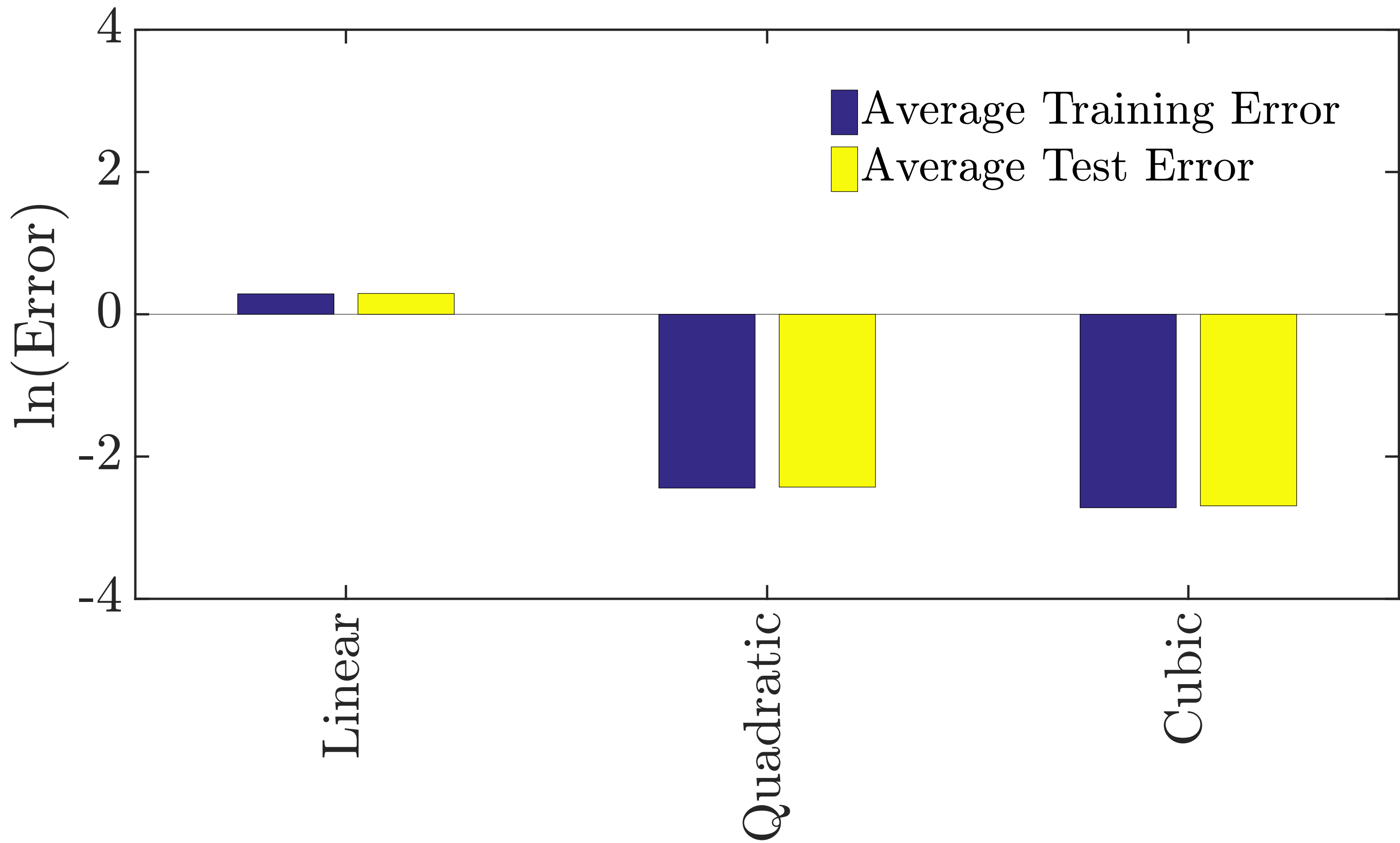

Supplement: Supplementary Information [file rspa20170117supp1.zip › Supplementary/Figures/erasing_regression_bar_chart-eps-converted-to.pdf]

- Simulation Reliability
- Cubic-Regression-Reliability

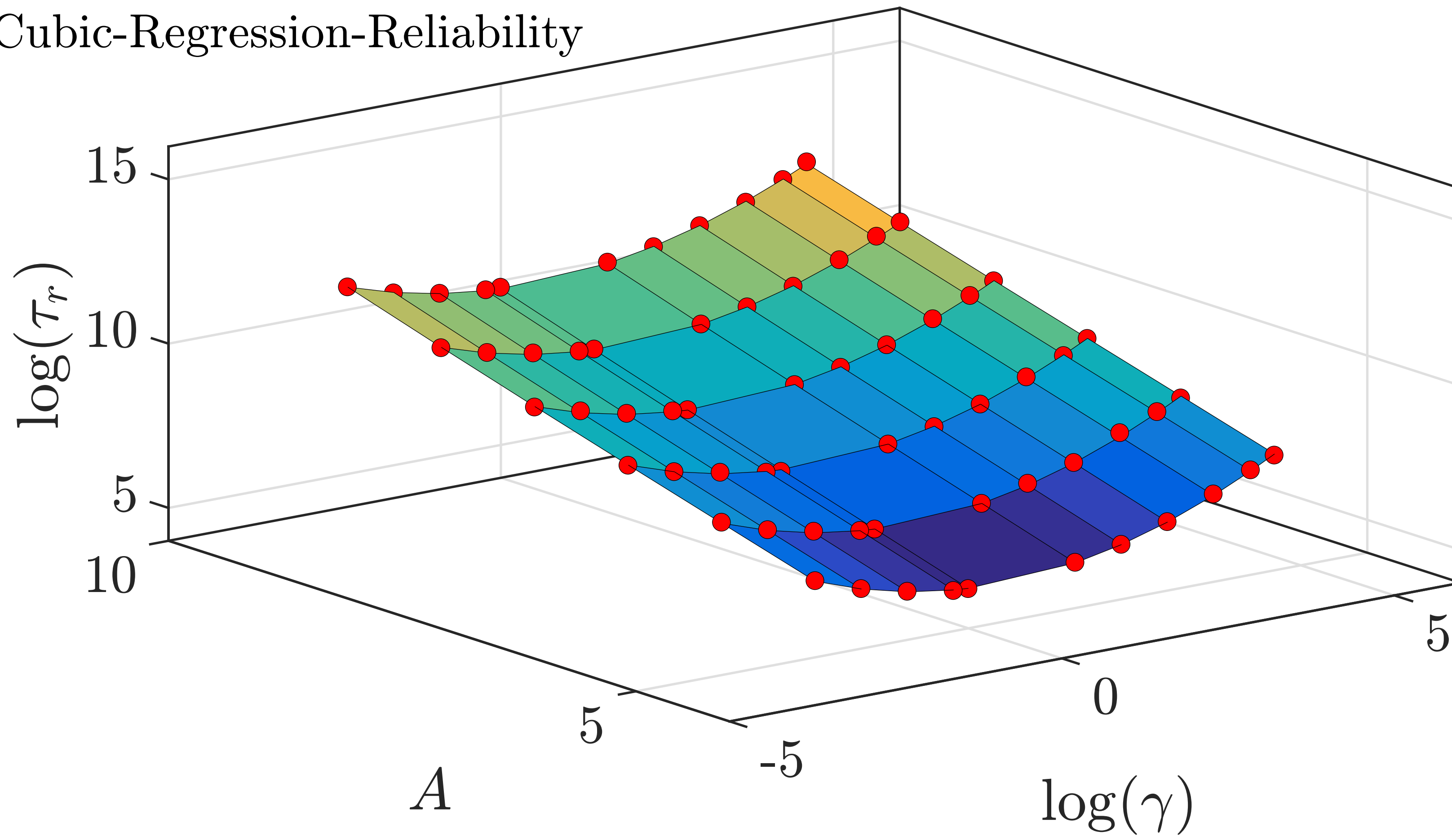

Supplement: Supplementary Information [file rspa20170117supp1.zip › Supplementary/Figures/cubic_regression_reliability-eps-converted-to.pdf]

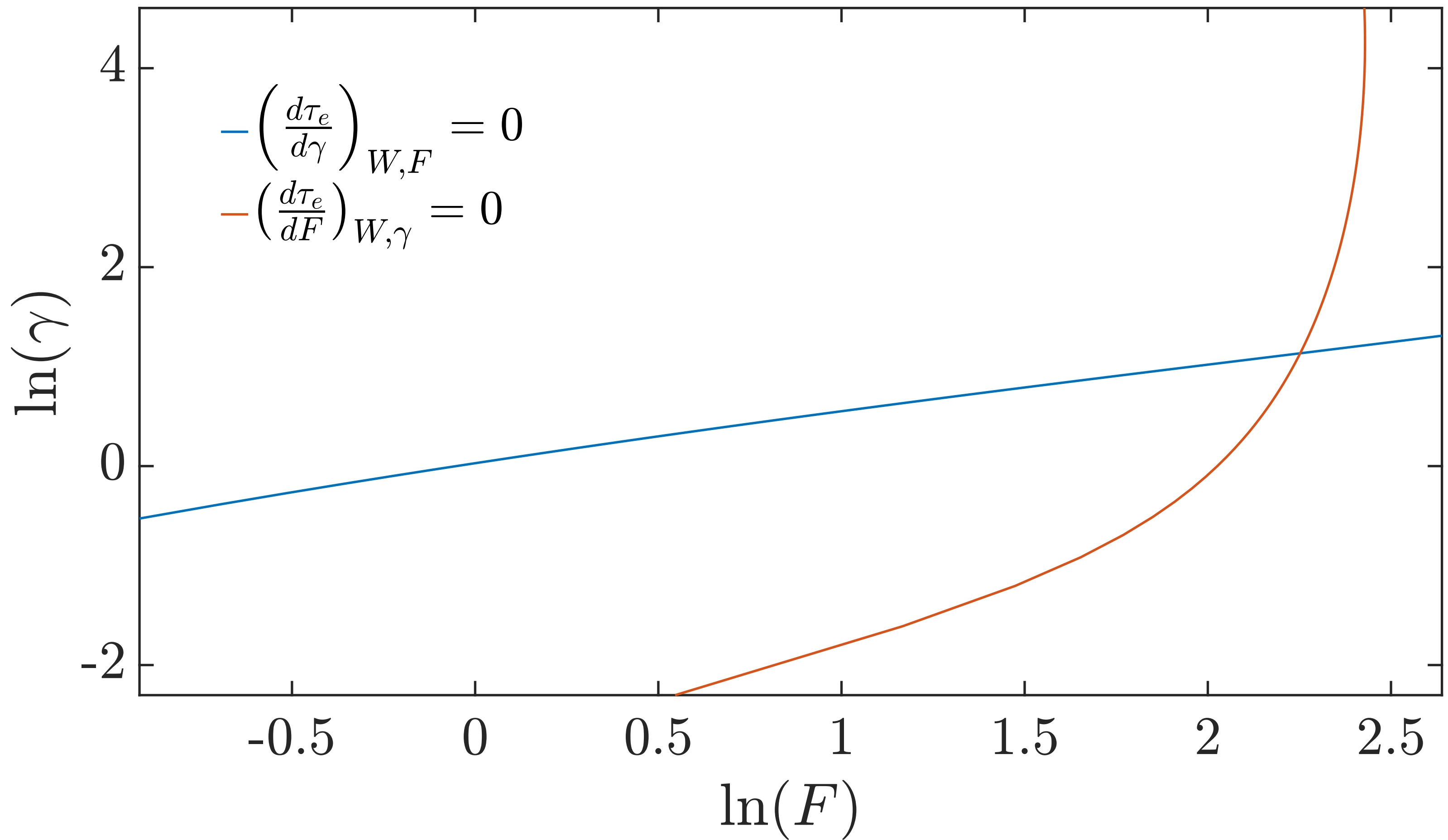

Supplement: Supplementary Information [file rspa20170117supp1.zip › Supplementary/Figures/single_local_minimum_W=19-eps-converted-to.pdf]

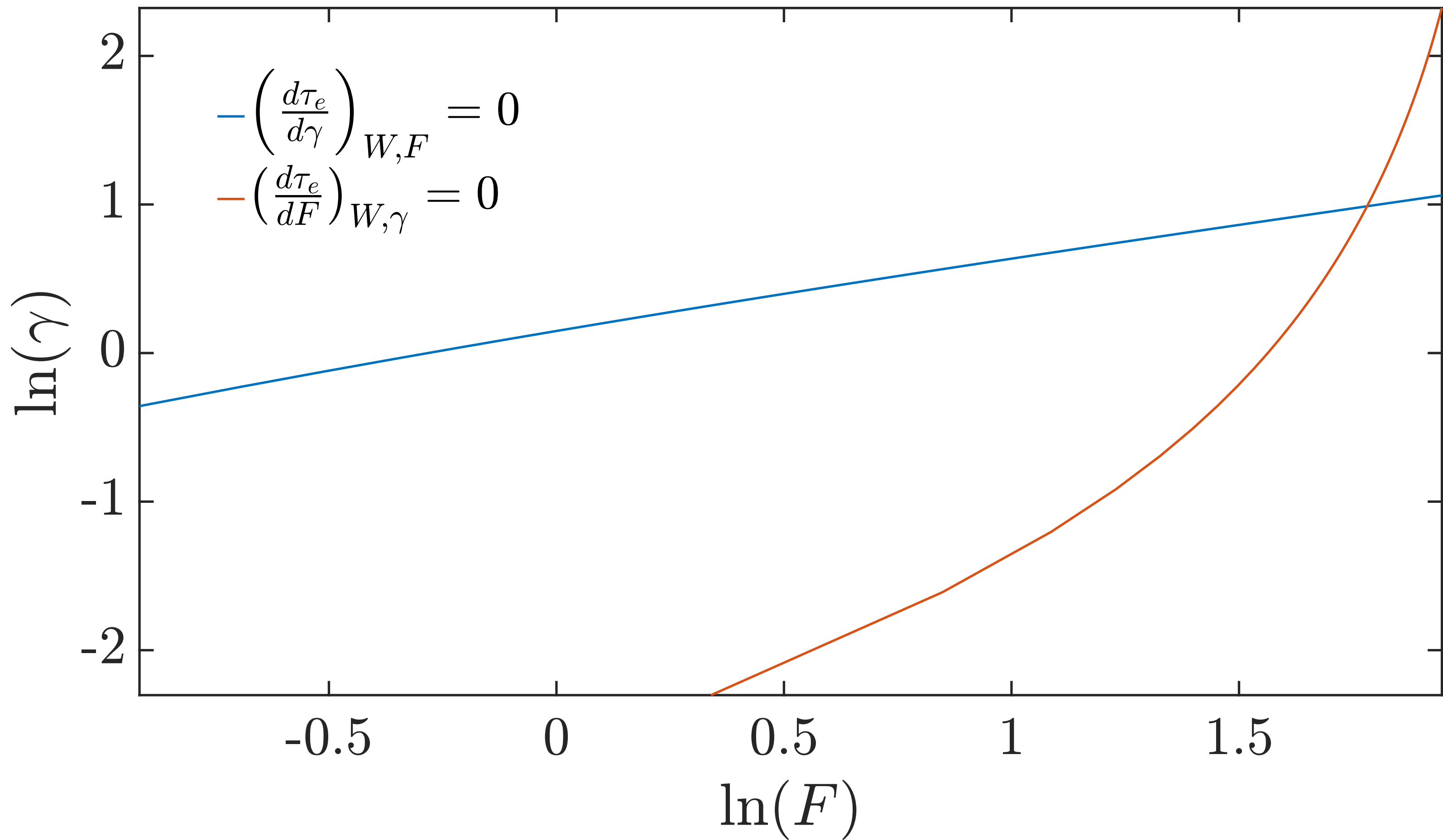

Supplement: Supplementary Information [file rspa20170117supp1.zip › Supplementary/Figures/single_local_minimum_W=12-eps-converted-to.pdf]

- Simulation Erasing
- Cubic-Regression-Erasing

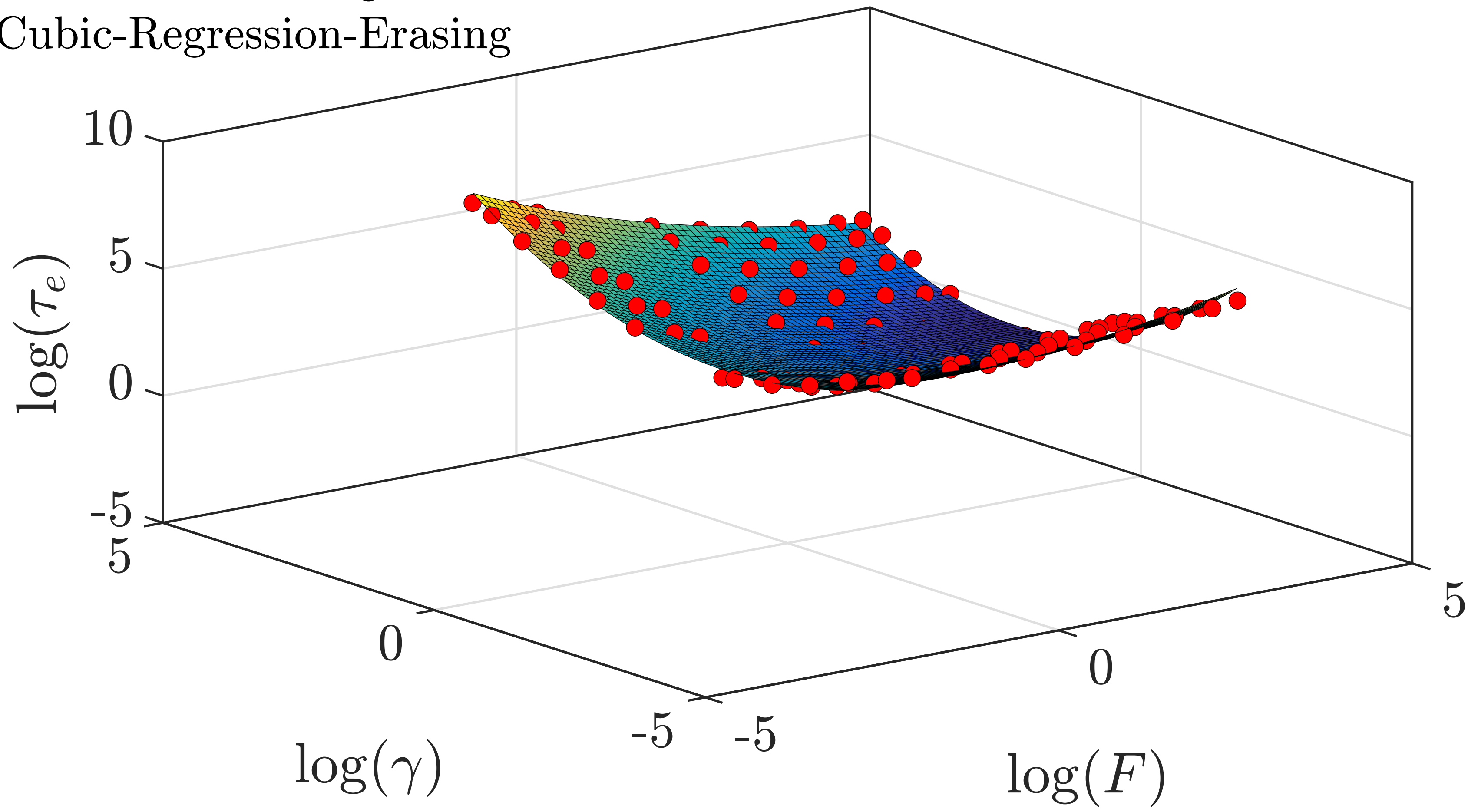

Supplement: Supplementary Information [file rspa20170117supp1.zip › Supplementary/Figures/cubic_regression_erasing-eps-converted-to.pdf]

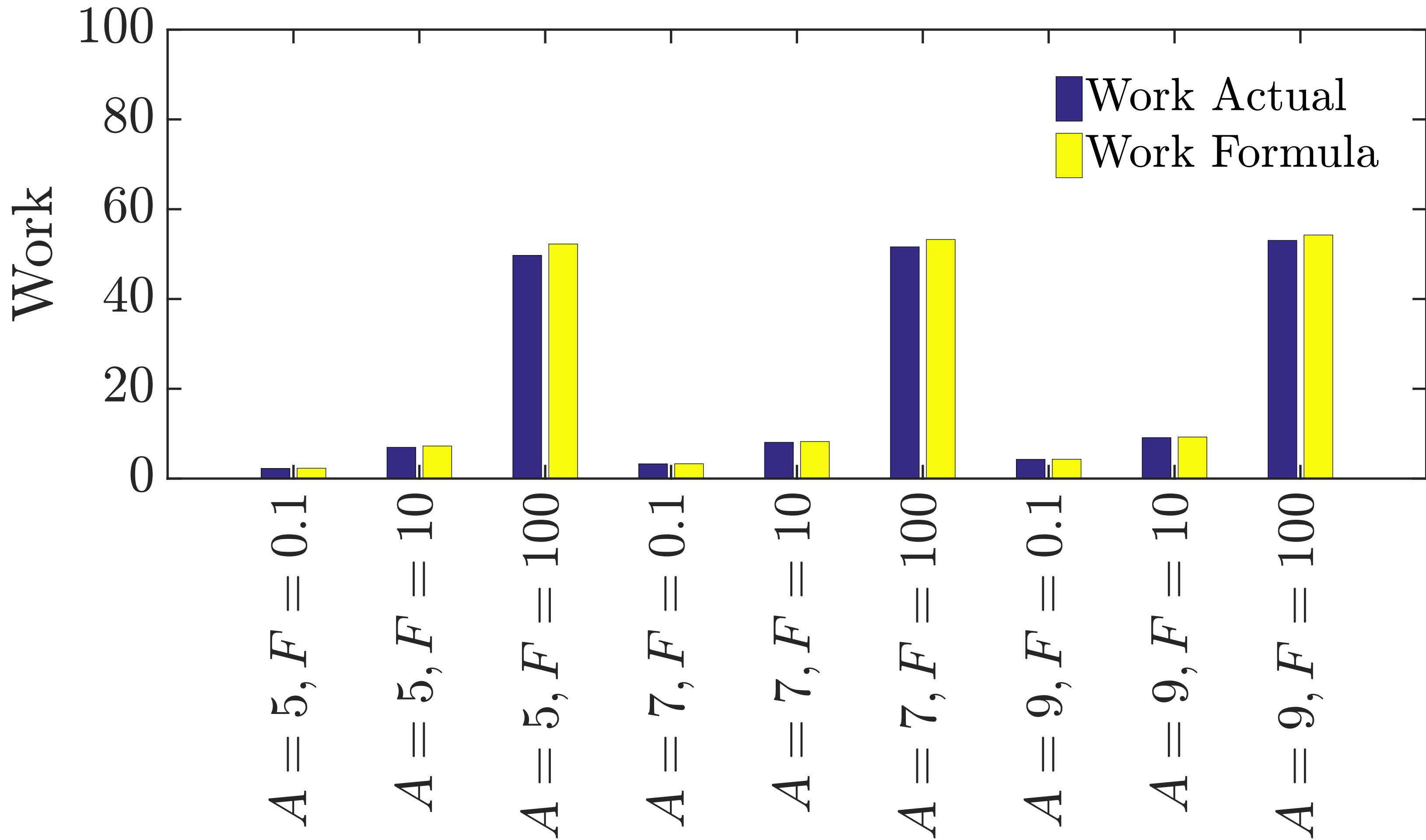

Supplement: Supplementary Information [file rspa20170117supp1.zip › Supplementary/Figures/work_accuracy-eps-converted-to.pdf]

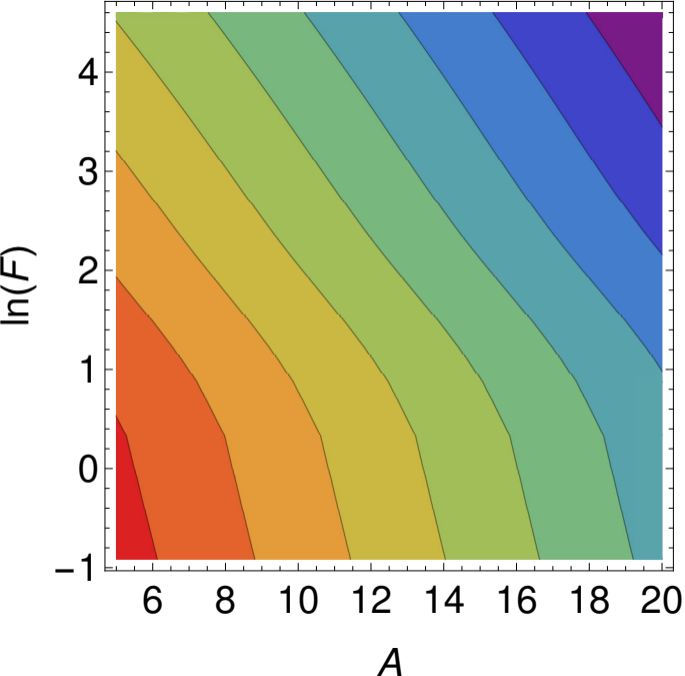

$\ln(\text{Fraction of recovered work})$

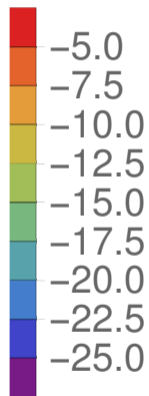

Supplement: Supplementary Information [file rspa20170117supp1.zip › Supplementary/Figures/recovered-eps-converted-to.pdf]

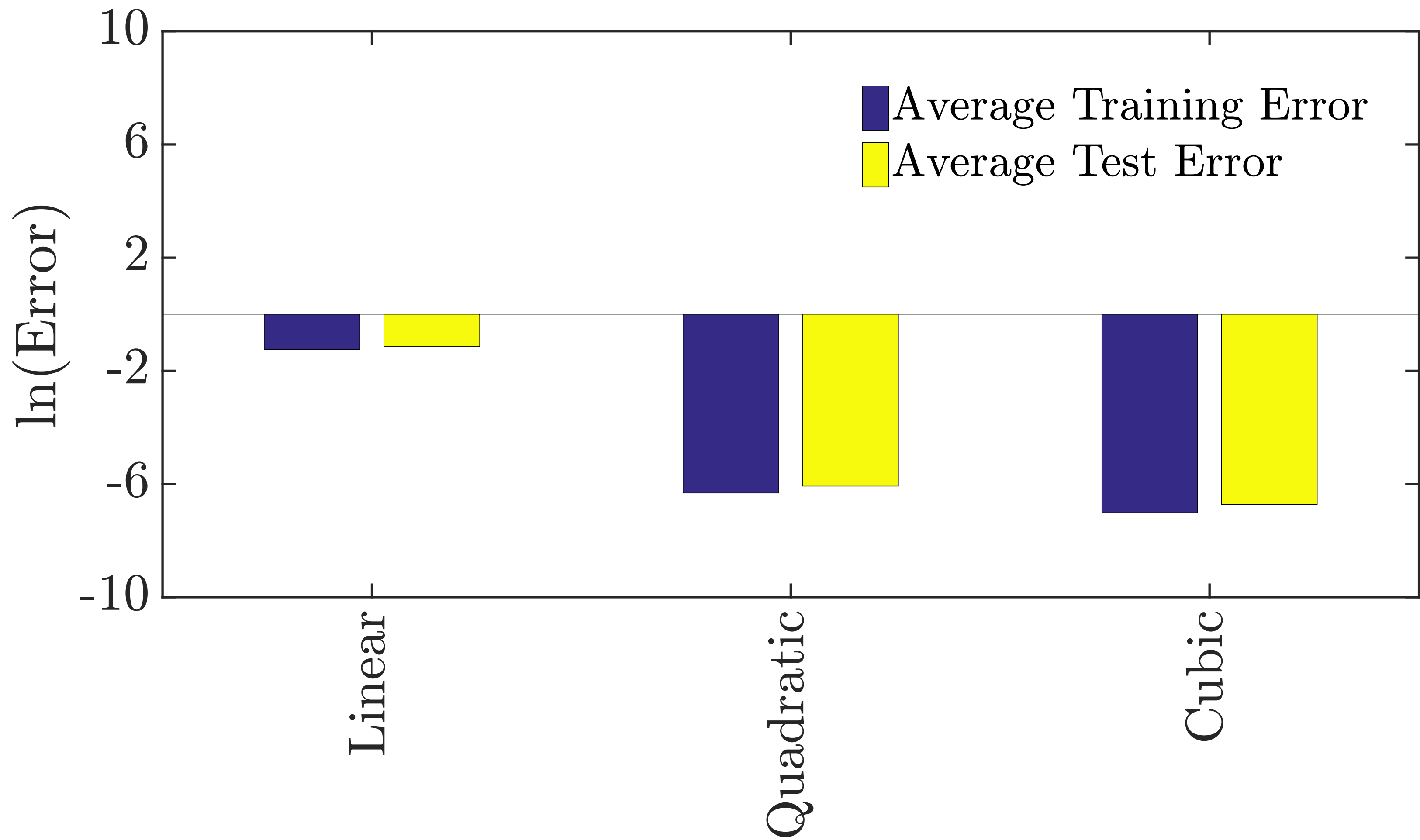

Supplement: Supplementary Information [file rspa20170117supp1.zip › Supplementary/Figures/reliability_regression_bar_chart-eps-converted-to.pdf]
